# Fast calculation of the quartet distance between trees of arbitrary degrees

**DOI:** 10.1186/1748-7188-1-16

**Published:** 2006-09-25

**Authors:** Chris Christiansen, Thomas Mailund, Christian NS Pedersen, Martin Randers, Martin Stig Stissing

**Affiliations:** 1 Department of Computer Science, University of Aarhus, Aabogade 34, DK-8200 Århus N, Denmark; 2 Department of Statistics, University of Oxford, 1 South Parks Road Oxford OX1 3TG, UK; 3 Bioinformatics Research Center, University of Aarhus, Høegh-Guldbergsgade 10, Bldg. 090, DK-8000 Århus C, Denmark

## Abstract

**Background:**

A number of algorithms have been developed for calculating the quartet distance between two evolutionary trees on the same set of species. The quartet distance is the number of quartets – sub-trees induced by four leaves – that differs between the trees. Mostly, these algorithms are restricted to work on binary trees, but recently we have developed algorithms that work on trees of arbitrary degree.

**Results:**

We present a fast algorithm for computing the quartet distance between trees of arbitrary degree. Given input trees *T *and *T'*, the algorithm runs in time *O*(*n *+ |*V*|·|*V'*| min{*id*, *id'*}) and space *O*(*n *+ |*V*|·|*V'*|), where *n *is the number of leaves in the two trees, *V *and *V *are the non-leaf nodes in *T *and *T'*, respectively, and *id *and *id' *are the maximal number of non-leaf nodes adjacent to a non-leaf node in *T *and *T'*, respectively. The fastest algorithms previously published for arbitrary degree trees run in *O*(*n*^3^) (independent of the degree of the tree) and *O*(|*V*|·|*V'*|·*id*·*id'*), respectively. We experimentally compare the algorithm with existing algorithms for computing the quartet distance for general trees.

**Conclusion:**

We present a new algorithm for computing the quartet distance between two trees of arbitrary degree. The new algorithm improves the asymptotic running time for computing the quartet distance, compared to previous methods, and experimental results indicate that the new method also performs significantly better in practice.

## Background

The evolutionary relationship for a set of species is conveniently described by a tree in which the leaves correspond to the species, and the internal nodes correspond to speciation events. The true evolutionary tree for a set of species is rarely known, so inferring it from obtainable information is of great interest. Many different methods have been developed for this, see e.g. [1] for an overview.

Different methods often yield different inferred trees for the same set of species, and even the same method can give rise to different evolutionary trees for the same set of species when applied to different information about the species. To study such differences in a systematic manner, one must be able to quantify differences between evolutionary trees using well-defined and efficient methods.

One approach for comparing evolutionary trees is to define a distance measure between trees and compare two trees by computing this distance. Several distance measures have been proposed, e.g. the symmetric difference metric [2], the nearest-neighbour interchange metric [3], the subtree transfer distance [4], the Robinson and Foulds distance [5], and the quartet distance [6]. Each distance measure has different properties and reflects different aspects of biology.

**Figure 1:**
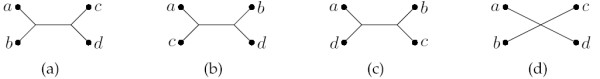
**The four possible quartet topologies**. The four possible quartet topologies of species *a*, *b*, *c*, *d*. Topologies (a): *ab*|*cd*, (b): *ac*|*bd*, and (c): *ad*|*bc *are *butterfly *quartets, while topology (d): ab×cd
 MathType@MTEF@5@5@+=feaafiart1ev1aaatCvAUfKttLearuWrP9MDH5MBPbIqV92AaeXatLxBI9gBaebbnrfifHhDYfgasaacH8akY=wiFfYdH8Gipec8Eeeu0xXdbba9frFj0=OqFfea0dXdd9vqai=hGuQ8kuc9pgc9s8qqaq=dirpe0xb9q8qiLsFr0=vr0=vr0dc8meaabaqaciaacaGaaeqabaqabeGadaaakeGadiahceaGeaapguaabeqaceaaaeaacqWGHbqyaeaacqWGIbGyaaGaey41aqBbaeqabiqaaaqaaiabdogaJbqaaiabdsgaKbaaaaa@3501@, is a *star *quartet.

This paper is concerned with calculating the quartet distance. A *quartet *is a set of four species, the *quartet topology *induced by an evolutionary tree is determined by the minimal topological subtree containing the four species. The four possible quartet topologies of four species are shown in Fig. 1. Given two evolutionary trees on the same set of *n *species, the *quartet distance *between them is the number of sets of four species for which the quartet topologies differ in the two trees.

Steel and Penny [7] pointed at Doucettes unpublished work [8] which presented an algorithm for computing the quartet distance in time *O*(*n*^3^), where *n *is the number of species. Bryant et al. in [9] presented an improved algorithm which computes the quartet distance in time *O*(*n*^2^) for binary trees. Brodal *et al*. in [10] showed how to compute the quartet distance in time *O*(*n *log *n*) considering binary trees. For arbitrary degree trees, the quartet distance can be calculated in time *O*(*n*^3^) or *O*(*n*^2^*d*^2^), where *d *is the maximum degree of any node in any of the two trees, as shown by Christiansen *et al*. [11].

## Results and discussion

In [11], we presented an algorithm for computing the quartet distance between trees of arbitrary degree. It runs in time *O*(*n*^2^*d*^2^) and space *O*(*n*^2^), where *n *is the number of leaves in each tree and *d *is the maximal degree found in either of the trees. In this paper, we present an improved algorithm running in time *O*(*n *+ |*V*||*V'*| min{*id*, *id'*}) and space *O*(*n *+ |*V*||*V'*|), where |*V| *and |*V'*| are the number of internal (non-leaf) nodes in the two input trees, and *id *and *id' *are the maximal degree of an internal node, when disregarding edges to leaves, in the two trees.

### Time analysis for different types of trees

The terms |*V*|, *id*, |*V'*| and *id' *are all clearly *O*(*n*), but on the other hand neither |*V*| and *id *nor |*V'*| and *id' *are independent. Intuitively, if there are a lot of internal nodes in a tree, they will not have a very large internal degree. We address in this section, how this dependency will affect the running time for different types on trees.

**Figure 2:**
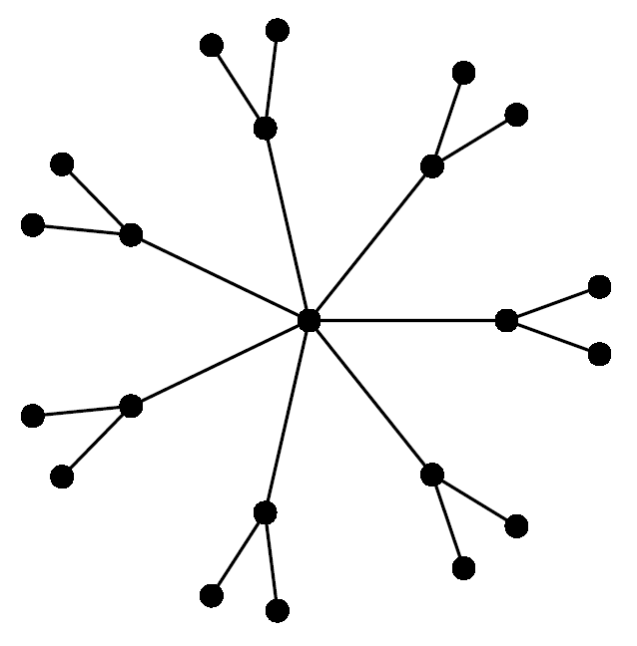
**A worst case input tree for the algorithm**. A tree with an internal node of degree n2
 MathType@MTEF@5@5@+=feaafiart1ev1aaatCvAUfKttLearuWrP9MDH5MBPbIqV92AaeXatLxBI9gBaebbnrfifHhDYfgasaacH8akY=wiFfYdH8Gipec8Eeeu0xXdbba9frFj0=OqFfea0dXdd9vqai=hGuQ8kuc9pgc9s8qqaq=dirpe0xb9q8qiLsFr0=vr0=vr0dc8meaabaqaciaacaGaaeqabaqabeGadaaakeaadaWcaaqaaiabd6gaUbqaaiabikdaYaaaaaa@2F13@, connected to n2
 MathType@MTEF@5@5@+=feaafiart1ev1aaatCvAUfKttLearuWrP9MDH5MBPbIqV92AaeXatLxBI9gBaebbnrfifHhDYfgasaacH8akY=wiFfYdH8Gipec8Eeeu0xXdbba9frFj0=OqFfea0dXdd9vqai=hGuQ8kuc9pgc9s8qqaq=dirpe0xb9q8qiLsFr0=vr0=vr0dc8meaabaqaciaacaGaaeqabaqabeGadaaakeaadaWcaaqaaiabd6gaUbqaaiabikdaYaaaaaa@2F13@ internal nodes of degree three each connected to two leaves. This tree has both a maximal degree of *O*(*n*) and at the same time *O*(*n*) inner nodes.

The worst theoretical running time of the algorithm for calculating the quartet distance presented above is *O*(*n*^3^). Consider a tree with an internal node of degree n2
 MathType@MTEF@5@5@+=feaafiart1ev1aaatCvAUfKttLearuWrP9MDH5MBPbIqV92AaeXatLxBI9gBaebbnrfifHhDYfgasaacH8akY=wiFfYdH8Gipec8Eeeu0xXdbba9frFj0=OqFfea0dXdd9vqai=hGuQ8kuc9pgc9s8qqaq=dirpe0xb9q8qiLsFr0=vr0=vr0dc8meaabaqaciaacaGaaeqabaqabeGadaaakeaadaWcaaqaaiabd6gaUbqaaiabikdaYaaaaaa@2F13@, connected to n2
 MathType@MTEF@5@5@+=feaafiart1ev1aaatCvAUfKttLearuWrP9MDH5MBPbIqV92AaeXatLxBI9gBaebbnrfifHhDYfgasaacH8akY=wiFfYdH8Gipec8Eeeu0xXdbba9frFj0=OqFfea0dXdd9vqai=hGuQ8kuc9pgc9s8qqaq=dirpe0xb9q8qiLsFr0=vr0=vr0dc8meaabaqaciaacaGaaeqabaqabeGadaaakeaadaWcaaqaaiabd6gaUbqaaiabikdaYaaaaaa@2F13@ internal nodes of degree three each connected to two leaves, see Fig. 2. Such a tree has *n *leaves, *O*(*n*) internal nodes and a maximal internal degree that is *O*(*n*). If the algorithm is run on two such trees, the running time will be *O*(*n*^3^). In *d*-ary trees (trees where all internal nodes have degree *d*) |*V*| = *O *(nd
 MathType@MTEF@5@5@+=feaafiart1ev1aaatCvAUfKttLearuWrP9MDH5MBPbIqV92AaeXatLxBI9gBaebbnrfifHhDYfgasaacH8akY=wiFfYdH8Gipec8Eeeu0xXdbba9frFj0=OqFfea0dXdd9vqai=hGuQ8kuc9pgc9s8qqaq=dirpe0xb9q8qiLsFr0=vr0=vr0dc8meaabaqaciaacaGaaeqabaqabeGadaaakeaadaWcaaqaaiabd6gaUbqaaiabdsgaKbaaaaa@2F72@), the time complexity of calculating the quartet distance will be *O *(n2d
 MathType@MTEF@5@5@+=feaafiart1ev1aaatCvAUfKttLearuWrP9MDH5MBPbIqV92AaeXatLxBI9gBaebbnrfifHhDYfgasaacH8akY=wiFfYdH8Gipec8Eeeu0xXdbba9frFj0=OqFfea0dXdd9vqai=hGuQ8kuc9pgc9s8qqaq=dirpe0xb9q8qiLsFr0=vr0=vr0dc8meaabaqaciaacaGaaeqabaqabeGadaaakeaadaWcaaqaaiabd6gaUnaaCaaaleqabaGaeGOmaidaaaGcbaGaemizaqgaaaaa@309B@).

The two cases above are somewhat extreme. The first case has a very large gap between the maximal and minimal degree of internal nodes, while the second has little or no gap. The theoretical performance of the algorithm on the two types of trees reflects this difference. Let *d*_*min *_= min{min_*v *_*d*_*v*_, min_*v' *_*d*_*v'*_}, be the minimal degree of any internal node in either tree, then each tree has *O*(ndmin
 MathType@MTEF@5@5@+=feaafiart1ev1aaatCvAUfKttLearuWrP9MDH5MBPbIqV92AaeXatLxBI9gBaebbnrfifHhDYfgasaacH8akY=wiFfYdH8Gipec8Eeeu0xXdbba9frFj0=OqFfea0dXdd9vqai=hGuQ8kuc9pgc9s8qqaq=dirpe0xb9q8qiLsFr0=vr0=vr0dc8meaabaqaciaacaGaaeqabaqabeGadaaakeaadaWcaaqaaiabd6gaUbqaaiabdsgaKnaaBaaaleaaieGacqWFTbqBcqWFPbqAcqWFUbGBaeqaaaaaaaa@33C0@) internal nodes and the time complexity is *O *(n2dmin2
 MathType@MTEF@5@5@+=feaafiart1ev1aaatCvAUfKttLearuWrP9MDH5MBPbIqV92AaeXatLxBI9gBaebbnrfifHhDYfgasaacH8akY=wiFfYdH8Gipec8Eeeu0xXdbba9frFj0=OqFfea0dXdd9vqai=hGuQ8kuc9pgc9s8qqaq=dirpe0xb9q8qiLsFr0=vr0=vr0dc8meaabaqaciaacaGaaeqabaqabeGadaaakeaadaWcaaqaaiabd6gaUnaaCaaaleqabaGaeGOmaidaaaGcbaGaemizaq2aa0baaSqaaGqaciab=1gaTjab=LgaPjab=5gaUbqaaiabikdaYaaaaaaaaa@35DC@ min{*id*, *id'*}). If min{*id*, *id'*} is *O*(dmin2
 MathType@MTEF@5@5@+=feaafiart1ev1aaatCvAUfKttLearuWrP9MDH5MBPbIqV92AaeXatLxBI9gBaebbnrfifHhDYfgasaacH8akY=wiFfYdH8Gipec8Eeeu0xXdbba9frFj0=OqFfea0dXdd9vqai=hGuQ8kuc9pgc9s8qqaq=dirpe0xb9q8qiLsFr0=vr0=vr0dc8meaabaqaciaacaGaaeqabaqabeGadaaakeaacqWGKbazdaqhaaWcbaacbiGae8xBa0Mae8xAaKMae8NBa4gabaGaeGOmaidaaaaa@333E@) the time usage of calculating the quartet distance will be *O *(*n*^2^). In the following section we will do practical verification of the theoretical results in this section.

### Experimental running times

**Figure 3:**
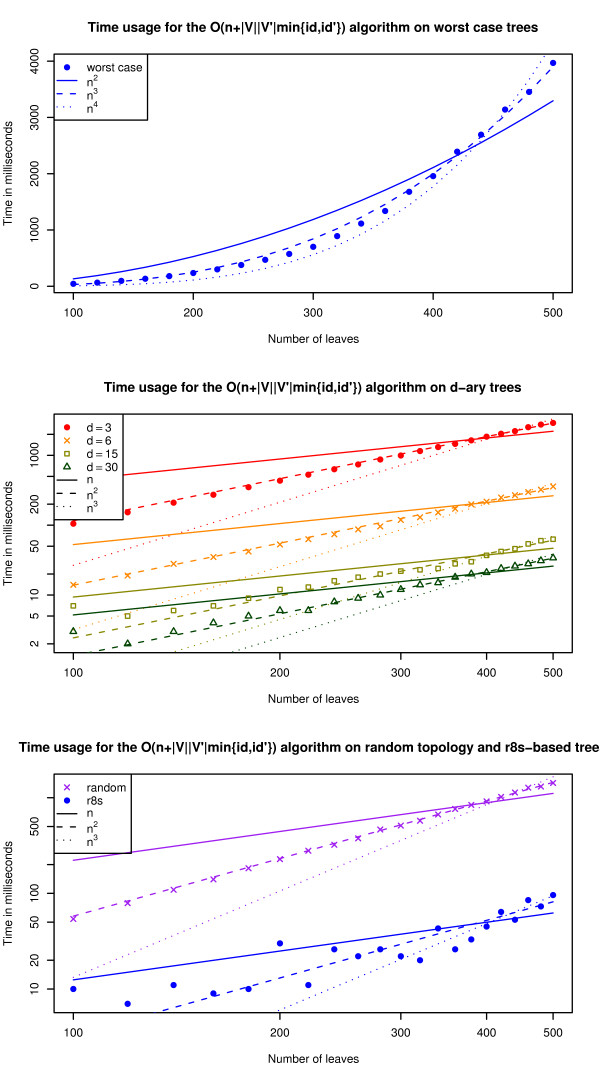
**Experimental running times**. The running time of the algorithm for worst case trees, *d*-ary trees and random trees. The lines plots the polynomials *c*. *n*^*i*^, where *c *is a fitted constant and *i *∈ [1, 4]. The two bottommost plots are in log-scale on both the x- and y-axis.

The graphs in Fig. 3 show the running time for comparing worst case trees (see Fig. 2), (*d*-ary trees and random trees. There are six types of (*d*-ary trees; binary, 6-ary, 15-ary, and 30-ary and two types of random trees; r8s-based (see [12]) and trees with random topologies. The trees generated by r8s are binary, but by contracting edges, we can get trees of arbitrary degree (contracting an edge *e *connecting nodes *u *and *v *means removing *u *and *e *and attaching the rest of *u*'s edges to *v*). Each edge is contracted with a probability that is inversely proportional with its length, i.e. a short edge has a higher probability of being contracted than a long edge. The trees with random topology are generated by adding leaves one by one, starting with a tree of size 2. A leaf can be added by attaching it to a random inner node or by spliting a random edge with a new node, to which the leaf is attached.

The running time for worst case input trees (as described in the previous section) is *O*(*n*^3^), because such trees have *O*(*n*) internal nodes and min{*id*, *id'*} is *O*(*n*). This is supported by the first graph in Fig. 3, which shows that the plot of the polynomial *n*^3 ^(representing the best sum-of-squares fit of the polynomial *c*·*n*^3 ^to the data-points) is closest to the plot of the running times with regard to slope.

The running time on the algorithm on *d*-ary trees is *O*(n2d
 MathType@MTEF@5@5@+=feaafiart1ev1aaatCvAUfKttLearuWrP9MDH5MBPbIqV92AaeXatLxBI9gBaebbnrfifHhDYfgasaacH8akY=wiFfYdH8Gipec8Eeeu0xXdbba9frFj0=OqFfea0dXdd9vqai=hGuQ8kuc9pgc9s8qqaq=dirpe0xb9q8qiLsFr0=vr0=vr0dc8meaabaqaciaacaGaaeqabaqabeGadaaakeaadaWcaaqaaiabd6gaUnaaCaaaleqabaGaeGOmaidaaaGcbaGaemizaqgaaaaa@309B@). The plots of the running times in the second graph are parallel, and one of them is plotted directly on top of a plot of the polynomial *n*^2 ^(here *c*·*n*^2 ^is fitted to the data-points for each *d *separately; the different colors match the *d *colors). This supports that they all have a running time of *O*(*n*^2^) for fixed *d*'s. The graph also shows that higher degrees give lower running times, which is also expected. The reason why the algorithm is more than twice as fast on 6-ary trees than it is on binary trees, is that the number of internal nodes in 6-ary trees is less than in binary trees, and even though |*V*| is *O*(*n*) in both cases, that difference has an impact on the running time. The last graph shows the running time of the algorithm on trees created as either random trees (each topology is equally likely) or trees simulated using r8s (with edge contraction as described above). We have no theoretical running time for this data, but the graphs show that the running time is *O*(*n*^2^). Even though the plotted data is only a small random sample, this indicates that many pairs of trees actually have the property that min{*id*, *id'*} is *O*(dmin2
 MathType@MTEF@5@5@+=feaafiart1ev1aaatCvAUfKttLearuWrP9MDH5MBPbIqV92AaeXatLxBI9gBaebbnrfifHhDYfgasaacH8akY=wiFfYdH8Gipec8Eeeu0xXdbba9frFj0=OqFfea0dXdd9vqai=hGuQ8kuc9pgc9s8qqaq=dirpe0xb9q8qiLsFr0=vr0=vr0dc8meaabaqaciaacaGaaeqabaqabeGadaaakeaacqWGKbazdaqhaaWcbaacbiGae8xBa0Mae8xAaKMae8NBa4gabaGaeGOmaidaaaaa@333E@). Therefore it is not unreasonable to expect that our algorithm runs in time *O*(*n*^2^) on trees used in practice. All experiments were performed on a standard PC (Pentium 4, 3 GHz, 1 Gb Ram) running Linux Fedora Core 3.

### Comparison with existing algorithms

**Figure 4:**
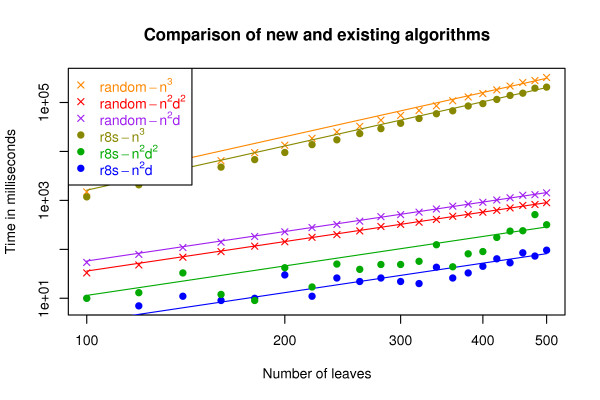
**Comparisons with earlier algorithms on random and r8s trees**. The running time for the new algorithm compared to the existing *O*(*n*^2^*d*^2^) and *O*(*n*^3^) time algorithms for random and r8s trees. The lines are fitted polynomial *c*. *n*^2^, for the case of the new algorithm (denoted *n*^2^*d *in the legend) and the *O*(*n*^2^*d*^2^) algorithm, and the polynomial *c*. *n*^3 ^for the *O*(*n*^3^) algorithm. The plot is in log-scale on both the x- and y-axis.

**Figure 5:**
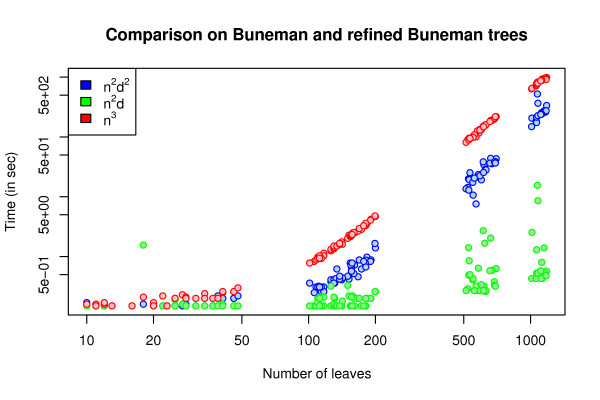
**Comparisons with earlier algorithms on Buneman and refined Buneman trees**. The running time for the new *O*(*n*^2^*d*) time algorithm compared to the existing *O*(*n*^2^*d*^2^) and *O*(*n*^3^) time algorithms on the Buneman and the refined Buneman trees for range of Pfam based distance matrices. The plot is in log-scale on both the x- and y-axis.

In Fig. 4 we compare the running time of the new algorithm with the *O*(*n*^2^*d*^2^) and *O*(*n*^3^) time algorithms from [11] on random and r8s simulated trees. In Fig. 5 we compare the running time of the new algorithm with the other two algorithms on Buneman and refined Buneman trees built for a range of Pfam [13] derived distance matrices using the tool in [14]. Buneman and refined Buneman trees are not binary unless this is well supported by the input distance matrix, and thus represent the kind of trees which can only be compared by methods which allow for trees of arbitrary degrees. In both experiments, the *O*(*n*^3^) time algorithm is slowest by a large margin for all plotted sizes of *n*. The new algorithm is consistently faster than the *O*(*n*^2^*d*^2^) time algorithm for the r8s (with edge contraction) simulated trees and for the Buneman and refined Buneman trees. For random trees the previous *O*(*n*^2^*d*^2^) time algorithm is slightly faster in practice. This difference is most likely caused by the additional overhead of precomputing the sums used by the new *O*(*n*^2^*d*) time algorithm compared to the previous *O*(*n*^2^*d*^2^) time algorithm in order to improve the asymptotic worst case running time (see method section). For trees of low degree, the overhead might dominate the factor *d *by which the worst case running time of the new algorithm is improved. The observed running times on random trees thus indicate that over selection of random trees consists of trees of low degree, whereas the r8s simulated, Buneman, and refined Buneman trees are trees with a few nodes of high degree which more than compensate for the additional overhead of dealing with nodes of low degree. In conclusion, we find that the experimental comparison of the new algorithm with the previously developed algorithms indicate that the new algorithm not only improves on the theoretical asymptotic running time, but also improves the running time in practice if the input trees contain a few nodes of high degree.

## Conclusion

We have constructed an algorithm for finding the quartet distance between two trees of arbitrary degree. It runs in time *O*(*n *+ |*V*||*V'*| min{*id*, *id'*}) and uses space *O*(*n *+ |*V*||*V'*|), where *n *is the number of leaves in the trees, |*V*| and |*V'*| are the number of internal nodes in the trees and *id *and *id' *are the maximal internal degree of internal nodes in input tree *T *and *T' *respectively. Internal degree of an internal node is the number of internal nodes connected to it, so neighbouring leaves do not add to this value. The values |*V*|, |*V'*|, *id *and *id' *are not independent, therefore we have investigated how the structure of the trees affect the running time of the algorithm. We show that the time used to count the butterfly quartets – topologies where one pair of the four leaves is separated from the other pair by an edge – is reduced to *O*(*n*^2^) when min{*id*, *id'*} = *O*(dmin2
 MathType@MTEF@5@5@+=feaafiart1ev1aaatCvAUfKttLearuWrP9MDH5MBPbIqV92AaeXatLxBI9gBaebbnrfifHhDYfgasaacH8akY=wiFfYdH8Gipec8Eeeu0xXdbba9frFj0=OqFfea0dXdd9vqai=hGuQ8kuc9pgc9s8qqaq=dirpe0xb9q8qiLsFr0=vr0=vr0dc8meaabaqaciaacaGaaeqabaqabeGadaaakeaacqWGKbazdaqhaaWcbaacbiGae8xBa0Mae8xAaKMae8NBa4gabaGaeGOmaidaaaaa@333E@), where *d*_*min *_is the minimal degree of all internal nodes in the trees. If the input trees are *d*-ary, that is all internal nodes have degree *d*, the running time is *O*(n2d
 MathType@MTEF@5@5@+=feaafiart1ev1aaatCvAUfKttLearuWrP9MDH5MBPbIqV92AaeXatLxBI9gBaebbnrfifHhDYfgasaacH8akY=wiFfYdH8Gipec8Eeeu0xXdbba9frFj0=OqFfea0dXdd9vqai=hGuQ8kuc9pgc9s8qqaq=dirpe0xb9q8qiLsFr0=vr0=vr0dc8meaabaqaciaacaGaaeqabaqabeGadaaakeaadaWcaaqaaiabd6gaUnaaCaaaleqabaGaeGOmaidaaaGcbaGaemizaqgaaaaa@309B@), excluding the time to find intersections. These theoretical running times have been validated by running a series of tests using a Java implementation of the algorithm, available at [15]. We also done a series of tests on random trees, trees generated by the program r8s, Buneman trees, and refined Buneman trees. Running the algorithm on these trees gives an impression on how it performs on trees used in practice. On both types of trees the running time appears to be *O*(*n*^2^). It is however still an open problem to develop an algorithm running in time *O*(*n*^2^)for all types of trees.

## Methods

Consider two input trees, and assume that a quartet has butterfly topology in both trees, i.e. that one pair of the four leaves is separated from the other pair by an edge in the tree in both trees. We say that the butterfly quartet is *shared*, if it has the same butterfly topology in both trees. Otherwise, we say that the butterfly quartet is *nonshared*. We let shared(*T*, *T'*) denote the number of butterflies shared between tree *T *and tree *T'*, i.e. the number of quartets that are butterflies with the same topology in tree *T *and tree *T'*, and let nonshared (*T*, *T'*) denote the number of quartets that are butterflies in both *T *and *T' *but with different topology. By our definition of *shared*, the number of butterfly quartets in a single tree can be stated as the number of butterfly quartets shared between the tree and itself, i.e. shared(*T*, *T*) or shared(*T'*, *T'*) for the number of butterfly quartets in *T *and *T' *respectively. (This notation also emphasizes that computing the number of butterfly quartets in a single tree by our algorithm is performed as a comparison of the tree against itself.)

In [11] we argue that the quartet distance between *T *and *T'*, qdist(*T*, *T'*), can be found by focusing only on the computation of the number of shared and nonshared butterfly quartets between two trees, i.e. it is unnecessary to consider non-butterfly quartets explicitely. More specifically, we show that:

qdist(T,T′)=shared(T,T)+shared(T′,T′)− 2⋅shared(T,T′)−nonshared(T,T′)     (1)
 MathType@MTEF@5@5@+=feaafiart1ev1aaatCvAUfKttLearuWrP9MDH5MBPbIqV92AaeXatLxBI9gBaebbnrfifHhDYfgasaacH8akY=wiFfYdH8Gipec8Eeeu0xXdbba9frFj0=OqFfea0dXdd9vqai=hGuQ8kuc9pgc9s8qqaq=dirpe0xb9q8qiLsFr0=vr0=vr0dc8meaabaqaciaacaGaaeqabaqabeGadaaakeaafaqadeGabaaabaGaeeyCaeNaeeizaqMaeeyAaKMaee4CamNaeeiDaqNaeiikaGIaemivaqLaeiilaWIafmivaqLbauaacqGGPaqkcqGH9aqpcqqGZbWCcqqGObaAcqqGHbqycqqGYbGCcqqGLbqzcqqGKbazcqGGOaakcqWGubavcqGGSaalcqWGubavcqGGPaqkcqGHRaWkcqqGZbWCcqqGObaAcqqGHbqycqqGYbGCcqqGLbqzcqqGKbazcqGGOaakcuWGubavgaqbaiabcYcaSiqbdsfauzaafaGaeiykaKcabaGaeyOeI0IaeeiiaaIaeGOmaiJaeyyXICTaee4CamNaeeiAaGMaeeyyaeMaeeOCaiNaeeyzauMaeeizaqMaeiikaGIaemivaqLaeiilaWIafmivaqLbauaacqGGPaqkcqGHsislcqqGUbGBcqqGVbWBcqqGUbGBcqqGZbWCcqqGObaAcqqGHbqycqqGYbGCcqqGLbqzcqqGKbazcqGGOaakcqWGubavcqGGSaalcuWGubavgaqbaiabcMcaPaaacaWLjaGaaCzcamaabmGabaGaeGymaedacaGLOaGaayzkaaaaaa@7CB7@

The proof of this formula is a follows. Let *Q *denote the number of quartets which have butterfly topology in *T *and non-butterfly topology in *T'*. Symmetrically, let *Q' *denote the number of quartets which have butterfly topology in *T' *and non-butterfly topology in T. A butterfly quartet in *T *is either a butterfly quartet in *T' *or a non-butterfly quartet in *T'*. The number of butterfly quartets in *T*, shared(*T*, *T*), can thus be expressed as the sum shared(*T*, *T'*) + nonshared(*T*, *T'*) + *Q*. Similarly, the sum shared (*T'*, *T'*) = *Q' *+ shared (*T*, *T'*) + nonshared (*T*, *T'*). It is now straightforward to verify that the righthand side of (1) adds up *Q *+ *Q' *+ nonshared(*T*, *T'*) which is the number of quartets where the quartet topologies differ in *T *and *T'*, i.e. qdist(*T*,*T'*).

In the section below, we describe how to use (1) to compute the quartet distance in time *O*(*n *+ |*V*||*V'*| min{*id*, *id'*}), more precisely *O*(*n *+ |*V*||*V'*|) for a preprocessing step, after which we can use *O*(|*V*||*V'*|) for calculating shared(*T*, *T'*), *O*(|*V*||*V'*|{*id*, *id'*}) for calculating nonshared(*T*,*T'*), *O*(|*V*|) for calculating shared(*T*,*T*) and *O*(|*V'*|) for calculating shared(*T'*,*T'*).

### Terminology

**Figure 6:**
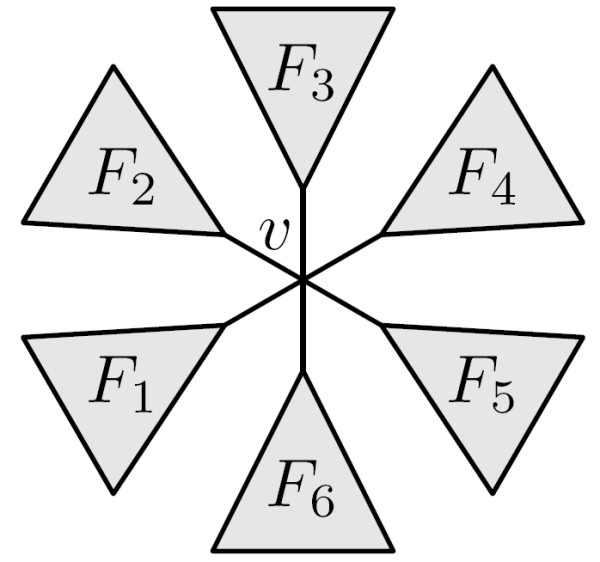
An internal node *v *∈ *T *with subtrees *F*_1_,...,*F*_*d*_, here *d*_*v *_= 6.

**Figure 7:**
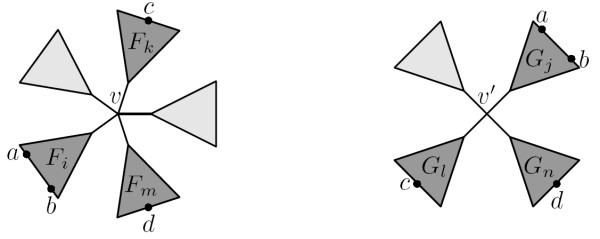
Internal nodes *v *∈ *T *and *v' *∈ *T'*, each claiming the quartet *ab*|*cd*.

Let *T *and *T' *be two unrooted trees. In this paper we will explicitly refer to the leaves of a tree as *leaves *and the non-leaf nodes as *internal node*. We will assume that *T *and *T' *each has *n *labelled leaves numbered 1,..., *n *such that the leaf numbered *x *in *T *has the same label as the leaf numbered *x *in *T'*. The leaf sets are denoted *L *and *L' *for *T *and *T' *respectively, note that *L *= *L'*. We will use *V *and *V' *to denote the internal nodes in *T *and *T' *respectively. The *degree *of an internal node *v *is the number of subtrees connected to it, and is denoted *d*_*v*_. The *internal degree *of an internal node *v*, *id*_*v*_, is the number of non-leaf subtrees connected to it. We will assume that no internal node in *T *and *T' *has degree two, and we will denote the maximal internal degree of all internal nodes in *T *and *T' *by *id *and *id' *respectively. Let *v *be an internal node in *T*, and let *F*_1_, ..., Fdv
 MathType@MTEF@5@5@+=feaafiart1ev1aaatCvAUfKttLearuWrP9MDH5MBPbIqV92AaeXatLxBI9gBaebbnrfifHhDYfgasaacH8akY=wiFfYdH8Gipec8Eeeu0xXdbba9frFj0=OqFfea0dXdd9vqai=hGuQ8kuc9pgc9s8qqaq=dirpe0xb9q8qiLsFr0=vr0=vr0dc8meaabaqaciaacaGaaeqabaqabeGadaaakeaacqWGgbGrdaWgaaWcbaGaemizaq2aaSbaaWqaaiabdAha2bqabaaaleqaaaaa@30EB@ be the subtrees connected to it, as shown in Fig. 6 We call these the subtrees *of v*. We say that *v claims *all butterfly quartets *ab*|*cd *where *a*,*b *∈ *F*_*i*_, *c *∈ *F*_*k *_and *d *∈ *F*_*m *_for *i *≠ *k *≠ *m *(see Fig. 7). With this definition, each butterfly quartet is claimed by exactly two internal nodes.

Adding the subscript *yz*|*w*|*x *to an internal node claiming the butterfly quartet *wx*|*yz*, indicates that the leaves *y *and *z *are found in a single subtree of the internal node, while the leaves *w *and *x *are found in different subtrees. For example, considering the quartet *ab*|*cd*, *v *and *v' *in Fig. 7 are written as *v*_*ab*|*c*|*d *_and v′ab|c|d
 MathType@MTEF@5@5@+=feaafiart1ev1aaatCvAUfKttLearuWrP9MDH5MBPbIqV92AaeXatLxBI9gBaebbnrfifHhDYfgasaacH8akY=wiFfYdH8Gipec8Eeeu0xXdbba9frFj0=OqFfea0dXdd9vqai=hGuQ8kuc9pgc9s8qqaq=dirpe0xb9q8qiLsFr0=vr0=vr0dc8meaabaqaciaacaGaaeqabaqabeGadaaakeaacuWG2bGDgaqbamaaBaaaleaacqWGHbqycqWGIbGycqGG8baFcqWGJbWycqGG8baFcqWGKbazaeqaaaaa@3691@.

**Figure 8:**
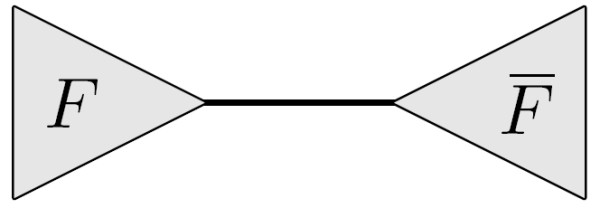
A rooted subtree *F*, and its complement rooted subtree F¯
 MathType@MTEF@5@5@+=feaafiart1ev1aaatCvAUfKttLearuWrP9MDH5MBPbIqV92AaeXatLxBI9gBaebbnrfifHhDYfgasaacH8akY=wiFfYdH8Gipec8Eeeu0xXdbba9frFj0=OqFfea0dXdd9vqai=hGuQ8kuc9pgc9s8qqaq=dirpe0xb9q8qiLsFr0=vr0=vr0dc8meaabaqaciaacaGaaeqabaqabeGadaaakeaacuWGgbGrgaqeaaaa@2DD9@.

Given a subtree *F *of *T*, and a subtree *G *of *T'*, we call the intersection *F *∩ *G *a *shared leaf set*, i.e. the set of leaves present in both *F *and *G*. The size of the shared leaf set, |*F *∩ *G*|, then denotes the number of leaves present in both *F *and *G*. The size of a single subtree *F *is similarly denoted |*F*|. We will use F¯
 MathType@MTEF@5@5@+=feaafiart1ev1aaatCvAUfKttLearuWrP9MDH5MBPbIqV92AaeXatLxBI9gBaebbnrfifHhDYfgasaacH8akY=wiFfYdH8Gipec8Eeeu0xXdbba9frFj0=OqFfea0dXdd9vqai=hGuQ8kuc9pgc9s8qqaq=dirpe0xb9q8qiLsFr0=vr0=vr0dc8meaabaqaciaacaGaaeqabaqabeGadaaakeaacuWGgbGrgaqeaaaa@2DD9@ to represent the subtree of *T *containing all leaves not in *F *and similarly for G¯
 MathType@MTEF@5@5@+=feaafiart1ev1aaatCvAUfKttLearuWrP9MDH5MBPbIqV92AaeXatLxBI9gBaebbnrfifHhDYfgasaacH8akY=wiFfYdH8Gipec8Eeeu0xXdbba9frFj0=OqFfea0dXdd9vqai=hGuQ8kuc9pgc9s8qqaq=dirpe0xb9q8qiLsFr0=vr0=vr0dc8meaabaqaciaacaGaaeqabaqabeGadaaakeaacuWGhbWrgaqeaaaa@2DDB@ and *G *in *T'*, see Fig. 8 for an example. Note that F¯
 MathType@MTEF@5@5@+=feaafiart1ev1aaatCvAUfKttLearuWrP9MDH5MBPbIqV92AaeXatLxBI9gBaebbnrfifHhDYfgasaacH8akY=wiFfYdH8Gipec8Eeeu0xXdbba9frFj0=OqFfea0dXdd9vqai=hGuQ8kuc9pgc9s8qqaq=dirpe0xb9q8qiLsFr0=vr0=vr0dc8meaabaqaciaacaGaaeqabaqabeGadaaakeaacuWGgbGrgaqeaaaa@2DD9@ and G¯
 MathType@MTEF@5@5@+=feaafiart1ev1aaatCvAUfKttLearuWrP9MDH5MBPbIqV92AaeXatLxBI9gBaebbnrfifHhDYfgasaacH8akY=wiFfYdH8Gipec8Eeeu0xXdbba9frFj0=OqFfea0dXdd9vqai=hGuQ8kuc9pgc9s8qqaq=dirpe0xb9q8qiLsFr0=vr0=vr0dc8meaabaqaciaacaGaaeqabaqabeGadaaakeaacuWGhbWrgaqeaaaa@2DDB@ are also subtrees of *T *and *T' *respectively, and thus |*F *∩ *G*|, |F¯
 MathType@MTEF@5@5@+=feaafiart1ev1aaatCvAUfKttLearuWrP9MDH5MBPbIqV92AaeXatLxBI9gBaebbnrfifHhDYfgasaacH8akY=wiFfYdH8Gipec8Eeeu0xXdbba9frFj0=OqFfea0dXdd9vqai=hGuQ8kuc9pgc9s8qqaq=dirpe0xb9q8qiLsFr0=vr0=vr0dc8meaabaqaciaacaGaaeqabaqabeGadaaakeaacuWGgbGrgaqeaaaa@2DD9@ ∩ *G*|, |*F *∩ G¯
 MathType@MTEF@5@5@+=feaafiart1ev1aaatCvAUfKttLearuWrP9MDH5MBPbIqV92AaeXatLxBI9gBaebbnrfifHhDYfgasaacH8akY=wiFfYdH8Gipec8Eeeu0xXdbba9frFj0=OqFfea0dXdd9vqai=hGuQ8kuc9pgc9s8qqaq=dirpe0xb9q8qiLsFr0=vr0=vr0dc8meaabaqaciaacaGaaeqabaqabeGadaaakeaacuWGhbWrgaqeaaaa@2DDB@| and |F¯
 MathType@MTEF@5@5@+=feaafiart1ev1aaatCvAUfKttLearuWrP9MDH5MBPbIqV92AaeXatLxBI9gBaebbnrfifHhDYfgasaacH8akY=wiFfYdH8Gipec8Eeeu0xXdbba9frFj0=OqFfea0dXdd9vqai=hGuQ8kuc9pgc9s8qqaq=dirpe0xb9q8qiLsFr0=vr0=vr0dc8meaabaqaciaacaGaaeqabaqabeGadaaakeaacuWGgbGrgaqeaaaa@2DD9@ ∩ G¯
 MathType@MTEF@5@5@+=feaafiart1ev1aaatCvAUfKttLearuWrP9MDH5MBPbIqV92AaeXatLxBI9gBaebbnrfifHhDYfgasaacH8akY=wiFfYdH8Gipec8Eeeu0xXdbba9frFj0=OqFfea0dXdd9vqai=hGuQ8kuc9pgc9s8qqaq=dirpe0xb9q8qiLsFr0=vr0=vr0dc8meaabaqaciaacaGaaeqabaqabeGadaaakeaacuWGhbWrgaqeaaaa@2DDB@| are all sizes of shared leaf sets between a *single *subtree from *T *and a *single *subtree from *T'*. In the presentation of the algorithms below, we will assume that we have access to |*F*|, |*G*| and |*F *∩ *G*| for all subtrees *F *of *T *and *G *of *T' *in time *O*(1). At the end of the section we will describe how this can be achieved by an *O*(*n*) time preprocessing step, which does not affect the asymptotic worst case running time of the presented algorithms.

### Counting shared butterfly quartets

For each pair of internal nodes *v*, *v' *from *T*, *T' *we want to count the number of shared butterfly quartets claimed by both internal nodes, shared(*v*, *v'*). Assume that *F*_1_, ..., Fdv
 MathType@MTEF@5@5@+=feaafiart1ev1aaatCvAUfKttLearuWrP9MDH5MBPbIqV92AaeXatLxBI9gBaebbnrfifHhDYfgasaacH8akY=wiFfYdH8Gipec8Eeeu0xXdbba9frFj0=OqFfea0dXdd9vqai=hGuQ8kuc9pgc9s8qqaq=dirpe0xb9q8qiLsFr0=vr0=vr0dc8meaabaqaciaacaGaaeqabaqabeGadaaakeaacqWGgbGrdaWgaaWcbaGaemizaq2aaSbaaWqaaiabdAha2bqabaaaleqaaaaa@30EB@ are subtrees of *v *and *G*_1_, ..., Gdv′
 MathType@MTEF@5@5@+=feaafiart1ev1aaatCvAUfKttLearuWrP9MDH5MBPbIqV92AaeXatLxBI9gBaebbnrfifHhDYfgasaacH8akY=wiFfYdH8Gipec8Eeeu0xXdbba9frFj0=OqFfea0dXdd9vqai=hGuQ8kuc9pgc9s8qqaq=dirpe0xb9q8qiLsFr0=vr0=vr0dc8meaabaqaciaacaGaaeqabaqabeGadaaakeaacqWGhbWrdaWgaaWcbaGaemizaq2aaSbaaWqaaiqbdAha2zaafaaabeaaaSqabaaaaa@30F9@ are subtrees of *v'*. We wish to count all quartets on the form *ab*|*cd *where *a*, *b *∈ *F*_*i *_∩ *G*_*j*_, *c *∈ *F*_*k *_∩ *G*_*l *_and *d *∈ *F*_*m *_∩ *G*_*n*_, *i *≠ *k *≠ *m*, *j *≠ *l *≠ *n *(see Fig. 7). Counting the possible combinations of *a *and *b *is expressed by the following double sum, which sums over all pairs of subtrees of *v *and *v'*:

∑i∑j(|Fi∩Gj|2)
 MathType@MTEF@5@5@+=feaafiart1ev1aaatCvAUfKttLearuWrP9MDH5MBPbIqV92AaeXatLxBI9gBaebbnrfifHhDYfgasaacH8akY=wiFfYdH8Gipec8Eeeu0xXdbba9frFj0=OqFfea0dXdd9vqai=hGuQ8kuc9pgc9s8qqaq=dirpe0xb9q8qiLsFr0=vr0=vr0dc8meaabaqaciaacaGaaeqabaqabeGadaaakeaadaaeqbqaamaaqafabaWaaeWaceaafaqabeGabaaabaGaeiiFaWNaemOray0aaSbaaSqaaiabdMgaPbqabaGccqGHPiYXcqWGhbWrdaWgaaWcbaGaemOAaOgabeaakiabcYha8bqaaiabikdaYaaaaiaawIcacaGLPaaaaSqaaiabdQgaQbqab0GaeyyeIuoaaSqaaiabdMgaPbqab0GaeyyeIuoaaaa@4022@

Given that *a *and *b *are in *F*_*i *_∩ *G*_*j*_, we need to find *c *and *d *in F¯
 MathType@MTEF@5@5@+=feaafiart1ev1aaatCvAUfKttLearuWrP9MDH5MBPbIqV92AaeXatLxBI9gBaebbnrfifHhDYfgasaacH8akY=wiFfYdH8Gipec8Eeeu0xXdbba9frFj0=OqFfea0dXdd9vqai=hGuQ8kuc9pgc9s8qqaq=dirpe0xb9q8qiLsFr0=vr0=vr0dc8meaabaqaciaacaGaaeqabaqabeGadaaakeaacuWGgbGrgaqeaaaa@2DD9@_*i *_∩ G¯
 MathType@MTEF@5@5@+=feaafiart1ev1aaatCvAUfKttLearuWrP9MDH5MBPbIqV92AaeXatLxBI9gBaebbnrfifHhDYfgasaacH8akY=wiFfYdH8Gipec8Eeeu0xXdbba9frFj0=OqFfea0dXdd9vqai=hGuQ8kuc9pgc9s8qqaq=dirpe0xb9q8qiLsFr0=vr0=vr0dc8meaabaqaciaacaGaaeqabaqabeGadaaakeaacuWGhbWrgaqeaaaa@2DDB@_*j*_. The number of possible choices of *c *and *d *is expressed by:

(|F¯i∩G¯j|2)
 MathType@MTEF@5@5@+=feaafiart1ev1aaatCvAUfKttLearuWrP9MDH5MBPbIqV92AaeXatLxBI9gBaebbnrfifHhDYfgasaacH8akY=wiFfYdH8Gipec8Eeeu0xXdbba9frFj0=OqFfea0dXdd9vqai=hGuQ8kuc9pgc9s8qqaq=dirpe0xb9q8qiLsFr0=vr0=vr0dc8meaabaqaciaacaGaaeqabaqabeGadaaakeaadaqadiqaauaabeqaceaaaeaacqGG8baFcuWGgbGrgaqeamaaBaaaleaacqWGPbqAaeqaaOGaeyykICSafm4raCKbaebadaWgaaWcbaGaemOAaOgabeaakiabcYha8bqaaiabikdaYaaaaiaawIcacaGLPaaaaaa@3954@

However when finding *c *and *d *in F¯
 MathType@MTEF@5@5@+=feaafiart1ev1aaatCvAUfKttLearuWrP9MDH5MBPbIqV92AaeXatLxBI9gBaebbnrfifHhDYfgasaacH8akY=wiFfYdH8Gipec8Eeeu0xXdbba9frFj0=OqFfea0dXdd9vqai=hGuQ8kuc9pgc9s8qqaq=dirpe0xb9q8qiLsFr0=vr0=vr0dc8meaabaqaciaacaGaaeqabaqabeGadaaakeaacuWGgbGrgaqeaaaa@2DD9@_*i *_∩ G¯
 MathType@MTEF@5@5@+=feaafiart1ev1aaatCvAUfKttLearuWrP9MDH5MBPbIqV92AaeXatLxBI9gBaebbnrfifHhDYfgasaacH8akY=wiFfYdH8Gipec8Eeeu0xXdbba9frFj0=OqFfea0dXdd9vqai=hGuQ8kuc9pgc9s8qqaq=dirpe0xb9q8qiLsFr0=vr0=vr0dc8meaabaqaciaacaGaaeqabaqabeGadaaakeaacuWGhbWrgaqeaaaa@2DDB@_*j*_, the condition that *c *and *d *must be in different subtrees is not satisfied. Therefore we subtract the number of times *c *and *d *are in the same subtree of *v *and *v'*:

∑k≠i(|Fk∩G¯j|2)+∑l≠j(|F¯i∩Gl|2)−∑k≠i∑l≠j(|Fk∩Gl|2)
 MathType@MTEF@5@5@+=feaafiart1ev1aaatCvAUfKttLearuWrP9MDH5MBPbIqV92AaeXatLxBI9gBaebbnrfifHhDYfgasaacH8akY=wiFfYdH8Gipec8Eeeu0xXdbba9frFj0=OqFfea0dXdd9vqai=hGuQ8kuc9pgc9s8qqaq=dirpe0xb9q8qiLsFr0=vr0=vr0dc8meaabaqaciaacaGaaeqabaqabeGadaaakeaadaaeqbqaamaabmGabaqbaeqabiqaaaqaaiabcYha8jabdAeagnaaBaaaleaacqWGRbWAaeqaaOGaeyykICSafm4raCKbaebadaWgaaWcbaGaemOAaOgabeaakiabcYha8bqaaiabikdaYaaaaiaawIcacaGLPaaacqGHRaWkdaaeqbqaamaabmGabaqbaeqabiqaaaqaaiabcYha8jqbdAeagzaaraWaaSbaaSqaaiabdMgaPbqabaGccqGHPiYXcqWGhbWrdaWgaaWcbaGaemiBaWgabeaakiabcYha8bqaaiabikdaYaaaaiaawIcacaGLPaaaaSqaaiabdYgaSjabgcMi5kabdQgaQbqab0GaeyyeIuoaaSqaaiabdUgaRjabgcMi5kabdMgaPbqab0GaeyyeIuoakiabgkHiTmaaqafabaWaaabuaeaadaqadiqaauaabeqaceaaaeaacqGG8baFcqWGgbGrdaWgaaWcbaGaem4AaSgabeaakiabgMIihlabdEeahnaaBaaaleaacqWGSbaBaeqaaOGaeiiFaWhabaGaeGOmaidaaaGaayjkaiaawMcaaaWcbaGaemiBaWMaeyiyIKRaemOAaOgabeqdcqGHris5aaWcbaGaem4AaSMaeyiyIKRaemyAaKgabeqdcqGHris5aaaa@6EC5@

Any pair in *F*_*k *_∩ *G*_*l *_is counted twice, once in |*F*_*k *_∩ G¯
 MathType@MTEF@5@5@+=feaafiart1ev1aaatCvAUfKttLearuWrP9MDH5MBPbIqV92AaeXatLxBI9gBaebbnrfifHhDYfgasaacH8akY=wiFfYdH8Gipec8Eeeu0xXdbba9frFj0=OqFfea0dXdd9vqai=hGuQ8kuc9pgc9s8qqaq=dirpe0xb9q8qiLsFr0=vr0=vr0dc8meaabaqaciaacaGaaeqabaqabeGadaaakeaacuWGhbWrgaqeaaaa@2DDB@_*j*_| and once in |F¯
 MathType@MTEF@5@5@+=feaafiart1ev1aaatCvAUfKttLearuWrP9MDH5MBPbIqV92AaeXatLxBI9gBaebbnrfifHhDYfgasaacH8akY=wiFfYdH8Gipec8Eeeu0xXdbba9frFj0=OqFfea0dXdd9vqai=hGuQ8kuc9pgc9s8qqaq=dirpe0xb9q8qiLsFr0=vr0=vr0dc8meaabaqaciaacaGaaeqabaqabeGadaaakeaacuWGgbGrgaqeaaaa@2DD9@_*i *_∩ *G*_*l*_|, therefore these pairs are subtracted once using the double sum above. (2) expresses the number of ways *c *and *d *can be found in different subtrees, given that a and *b *are found in *F*_*i *_∩ *G*_*j*_:

(|F¯i∩G¯j|2)−∑k≠i(|Fk∩G¯j|2)−∑l≠j(|F¯i∩Gl|2)+∑k≠i∑l≠j(|Fk∩Gl|2)     (2)
 MathType@MTEF@5@5@+=feaafiart1ev1aaatCvAUfKttLearuWrP9MDH5MBPbIqV92AaeXatLxBI9gBaebbnrfifHhDYfgasaacH8akY=wiFfYdH8Gipec8Eeeu0xXdbba9frFj0=OqFfea0dXdd9vqai=hGuQ8kuc9pgc9s8qqaq=dirpe0xb9q8qiLsFr0=vr0=vr0dc8meaabaqaciaacaGaaeqabaqabeGadaaakeaadaqadiqaauaabeqaceaaaeaacqGG8baFcuWGgbGrgaqeamaaBaaaleaacqWGPbqAaeqaaOGaeyykICSafm4raCKbaebadaWgaaWcbaGaemOAaOgabeaakiabcYha8bqaaiabikdaYaaaaiaawIcacaGLPaaacqGHsisldaaeqbqaamaabmGabaqbaeqabiqaaaqaaiabcYha8jabdAeagnaaBaaaleaacqWGRbWAaeqaaOGaeyykICSafm4raCKbaebadaWgaaWcbaGaemOAaOgabeaakiabcYha8bqaaiabikdaYaaaaiaawIcacaGLPaaaaSqaaiabdUgaRjabgcMi5kabdMgaPbqab0GaeyyeIuoakiabgkHiTmaaqafabaWaaeWaceaafaqabeGabaaabaGaeiiFaWNafmOrayKbaebadaWgaaWcbaGaemyAaKgabeaakiabgMIihlabdEeahnaaBaaaleaacqWGSbaBaeqaaOGaeiiFaWhabaGaeGOmaidaaaGaayjkaiaawMcaaaWcbaGaemiBaWMaeyiyIKRaemOAaOgabeqdcqGHris5aOGaey4kaSYaaabuaeaadaaeqbqaamaabmGabaqbaeqabiqaaaqaaiabcYha8jabdAeagnaaBaaaleaacqWGRbWAaeqaaOGaeyykICSaem4raC0aaSbaaSqaaiabdYgaSbqabaGccqGG8baFaeaacqaIYaGmaaaacaGLOaGaayzkaaaaleaacqWGSbaBcqGHGjsUcqWGQbGAaeqaniabggHiLdaaleaacqWGRbWAcqGHGjsUcqWGPbqAaeqaniabggHiLdGccaWLjaGaaCzcamaabmGabaGaeGOmaidacaGLOaGaayzkaaaaaa@802F@

We can now compute the number of shared butterfly quartets between two internal nodes, ie. the number of butterfly quartets claimed by both internal nodes with the same topology:

shared(v,v′)=∑i∑j(|Fi∩Gj|2)((|F¯i∩G¯j|2)−∑k≠i(|Fk∩G¯j|2)−∑l≠j(|F¯i∩Gl|2)+∑k≠i∑l≠j(|Fk∩Gl|2))     (3)
 MathType@MTEF@5@5@+=feaafiart1ev1aaatCvAUfKttLearuWrP9MDH5MBPbIqV92AaeXatLxBI9gBaebbnrfifHhDYfgasaacH8akY=wiFfYdH8Gipec8Eeeu0xXdbba9frFj0=OqFfea0dXdd9vqai=hGuQ8kuc9pgc9s8qqaq=dirpe0xb9q8qiLsFr0=vr0=vr0dc8meaabaqaciaacaGaaeqabaqabeGadaaakeaafaqaceGabaaabaGaee4CamNaeeiAaGMaeeyyaeMaeeOCaiNaeeyzauMaeeizaq2aaeWaceaacqWG2bGDcqGGSaalcuWG2bGDgaqbaaGaayjkaiaawMcaaiabg2da9maaqafabaWaaabuaeaadaqadiqaauaabeqaceaaaeaacqGG8baFcqWGgbGrdaWgaaWcbaGaemyAaKgabeaakiabgMIihlabdEeahnaaBaaaleaacqWGQbGAaeqaaOGaeiiFaWhabaGaeGOmaidaaaGaayjkaiaawMcaaaWcbaGaemOAaOgabeqdcqGHris5aaWcbaGaemyAaKgabeqdcqGHris5aOWaaeqaceaadaqadiqaauaabeqaceaaaeaacqGG8baFcuWGgbGrgaqeamaaBaaaleaacqWGPbqAaeqaaOGaeyykICSafm4raCKbaebadaWgaaWcbaGaemOAaOgabeaakiabcYha8bqaaiabikdaYaaaaiaawIcacaGLPaaacqGHsisldaaeqbqaamaabmGabaqbaeqabiqaaaqaaiabcYha8jabdAeagnaaBaaaleaacqWGRbWAaeqaaOGaeyykICSafm4raCKbaebadaWgaaWcbaGaemOAaOgabeaakiabcYha8bqaaiabikdaYaaaaiaawIcacaGLPaaaaSqaaiabdUgaRjabgcMi5kabdMgaPbqab0GaeyyeIuoaaOGaayjkaaaabaWaaeGaceaacqGHsisldaaeqbqaamaabmGabaqbaeqabiqaaaqaaiabcYha8jqbdAeagzaaraWaaSbaaSqaaiabdMgaPbqabaGccqGHPiYXcqWGhbWrdaWgaaWcbaGaemiBaWgabeaakiabcYha8bqaaiabikdaYaaaaiaawIcacaGLPaaacqGHRaWkdaaeqbqaamaaqafabaWaaeWaceaafaqabeGabaaabaGaeiiFaWNaemOray0aaSbaaSqaaiabdUgaRbqabaGccqGHPiYXcqWGhbWrdaWgaaWcbaGaemiBaWgabeaakiabcYha8bqaaiabikdaYaaaaiaawIcacaGLPaaaaSqaaiabdYgaSjabgcMi5kabdQgaQbqab0GaeyyeIuoaaSqaaiabdUgaRjabgcMi5kabdMgaPbqab0GaeyyeIuoaaSqaaiabdYgaSjabgcMi5kabdQgaQbqab0GaeyyeIuoaaOGaayzkaaaaaiaaxMaacaWLjaWaaeWaceaacqaIZaWmaiaawIcacaGLPaaaaaa@A3C6@

If the trees, *T *and *T'*, have a shared quartet *ab*|*cd*, then there are two internal nodes in each tree that claims this quartet: *v*_*ab*|*c*|*d *_and *v*_*cd*|*a*|*b *_in *T *and v′ab|c|d
 MathType@MTEF@5@5@+=feaafiart1ev1aaatCvAUfKttLearuWrP9MDH5MBPbIqV92AaeXatLxBI9gBaebbnrfifHhDYfgasaacH8akY=wiFfYdH8Gipec8Eeeu0xXdbba9frFj0=OqFfea0dXdd9vqai=hGuQ8kuc9pgc9s8qqaq=dirpe0xb9q8qiLsFr0=vr0=vr0dc8meaabaqaciaacaGaaeqabaqabeGadaaakeaacuWG2bGDgaqbamaaBaaaleaacqWGHbqycqWGIbGycqGG8baFcqWGJbWycqGG8baFcqWGKbazaeqaaaaa@3691@ and v′cd|a|b
 MathType@MTEF@5@5@+=feaafiart1ev1aaatCvAUfKttLearuWrP9MDH5MBPbIqV92AaeXatLxBI9gBaebbnrfifHhDYfgasaacH8akY=wiFfYdH8Gipec8Eeeu0xXdbba9frFj0=OqFfea0dXdd9vqai=hGuQ8kuc9pgc9s8qqaq=dirpe0xb9q8qiLsFr0=vr0=vr0dc8meaabaqaciaacaGaaeqabaqabeGadaaakeaacuWG2bGDgaqbamaaBaaaleaacqWGJbWycqWGKbazcqGG8baFcqWGHbqycqGG8baFcqWGIbGyaeqaaaaa@3691@ in *T'*. Since both shared(*v*_*ab*|*c*|*d*_, v′ab|c|d
 MathType@MTEF@5@5@+=feaafiart1ev1aaatCvAUfKttLearuWrP9MDH5MBPbIqV92AaeXatLxBI9gBaebbnrfifHhDYfgasaacH8akY=wiFfYdH8Gipec8Eeeu0xXdbba9frFj0=OqFfea0dXdd9vqai=hGuQ8kuc9pgc9s8qqaq=dirpe0xb9q8qiLsFr0=vr0=vr0dc8meaabaqaciaacaGaaeqabaqabeGadaaakeaacuWG2bGDgaqbamaaBaaaleaacqWGHbqycqWGIbGycqGG8baFcqWGJbWycqGG8baFcqWGKbazaeqaaaaa@3691@) and shared(*v*_*cd*|*a*|*b*_, v′cd|a|b
 MathType@MTEF@5@5@+=feaafiart1ev1aaatCvAUfKttLearuWrP9MDH5MBPbIqV92AaeXatLxBI9gBaebbnrfifHhDYfgasaacH8akY=wiFfYdH8Gipec8Eeeu0xXdbba9frFj0=OqFfea0dXdd9vqai=hGuQ8kuc9pgc9s8qqaq=dirpe0xb9q8qiLsFr0=vr0=vr0dc8meaabaqaciaacaGaaeqabaqabeGadaaakeaacuWG2bGDgaqbamaaBaaaleaacqWGJbWycqWGKbazcqGG8baFcqWGHbqycqGG8baFcqWGIbGyaeqaaaaa@3691@) will count the quartet, the total number of shared quartets between the two trees is:

shared(T,T′)=12∑v∈T∑v′∈T′shared(v,v′)
 MathType@MTEF@5@5@+=feaafiart1ev1aaatCvAUfKttLearuWrP9MDH5MBPbIqV92AaeXatLxBI9gBaebbnrfifHhDYfgasaacH8akY=wiFfYdH8Gipec8Eeeu0xXdbba9frFj0=OqFfea0dXdd9vqai=hGuQ8kuc9pgc9s8qqaq=dirpe0xb9q8qiLsFr0=vr0=vr0dc8meaabaqaciaacaGaaeqabaqabeGadaaakeaacqqGZbWCcqqGObaAcqqGHbqycqqGYbGCcqqGLbqzcqqGKbazcqGGOaakcqWGubavcqGGSaalcuWGubavgaqbaiabcMcaPiabg2da9maalaaabaGaeGymaedabaGaeGOmaidaamaaqafabaWaaabuaeaacqqGZbWCcqqGObaAcqqGHbqycqqGYbGCcqqGLbqzcqqGKbazcqGGOaakcqWG2bGDcqGGSaalcuWG2bGDgaqbaiabcMcaPaWcbaGafmODayNbauaacqGHiiIZcuWGubavgaqbaaqab0GaeyyeIuoaaSqaaiabdAha2jabgIGiolabdsfaubqab0GaeyyeIuoaaaa@570E@

It is straightforward to observe that calculating shared(*v*, *v'*) using a direct computation of (3) takes time *O*(dv2dv′2
 MathType@MTEF@5@5@+=feaafiart1ev1aaatCvAUfKttLearuWrP9MDH5MBPbIqV92AaeXatLxBI9gBaebbnrfifHhDYfgasaacH8akY=wiFfYdH8Gipec8Eeeu0xXdbba9frFj0=OqFfea0dXdd9vqai=hGuQ8kuc9pgc9s8qqaq=dirpe0xb9q8qiLsFr0=vr0=vr0dc8meaabaqaciaacaGaaeqabaqabeGadaaakeaacqWGKbazdaqhaaWcbaGaemODayhabaGaeGOmaidaaOGaemizaq2aa0baaSqaaiqbdAha2zaafaaabaGaeGOmaidaaaaa@348C@). It is however not necessary for shared(*v*, *v'*) to sum over all subtrees of *v *and *v'*. Since each term in the sums involves taking a 2-subset from a shared leaf set, we need only to consider subtrees that are not leaves. This reduces the running time to *O*(idv2idv′2
 MathType@MTEF@5@5@+=feaafiart1ev1aaatCvAUfKttLearuWrP9MDH5MBPbIqV92AaeXatLxBI9gBaebbnrfifHhDYfgasaacH8akY=wiFfYdH8Gipec8Eeeu0xXdbba9frFj0=OqFfea0dXdd9vqai=hGuQ8kuc9pgc9s8qqaq=dirpe0xb9q8qiLsFr0=vr0=vr0dc8meaabaqaciaacaGaaeqabaqabeGadaaakeaacqWGPbqAcqWGKbazdaqhaaWcbaGaemODayhabaGaeGOmaidaaOGaemyAaKMaemizaq2aa0baaSqaaiqbdAha2zaafaaabaGaeGOmaidaaaaa@3742@). This running time can be improved even more, we start by expressing (2) in a different way:

(|F¯i∩G¯j|2)︸I−∑k≠i(|Fk∩G¯j|2)︸II−∑l≠j(|F¯i∩Gl|2)︸III+∑k≠i∑l≠j(|Fk∩Gl|2)︸IV=(|F¯i∩G¯j|2)︸I−∑k(|Fk∩G¯j|2)+(|Fi∩G¯j|2)︸II−∑l(|F¯i∩Gl|2)+(|F¯i∩Gj|2)︸III+∑k∑l(|Fk∩Gl|2)−∑k(|Fk∩Gj|2)−∑l(|Fi∩Gl|2)+(|Fi∩Gj|2)︸IV     (4)
 MathType@MTEF@5@5@+=feaafiart1ev1aaatCvAUfKttLearuWrP9MDH5MBPbIqV92AaeXatLxBI9gBaebbnrfifHhDYfgasaacH8akY=wiFfYdH8Gipec8Eeeu0xXdbba9frFj0=OqFfea0dXdd9vqai=hGuQ8kuc9pgc9s8qqaq=dirpe0xb9q8qiLsFr0=vr0=vr0dc8meaabaqaciaacaGaaeqabaqabeGadaaakeaafaqabeWabaaabaWaaGbaaeaadaqadiqaauaabeqaceaaaeaacqGG8baFcuWGgbGrgaqeamaaBaaaleaacqWGPbqAaeqaaOGaeyykICSafm4raCKbaebadaWgaaWcbaGaemOAaOgabeaakiabcYha8bqaaiabikdaYaaaaiaawIcacaGLPaaaaSqaaiabdMeajbGccaGL44pacqGHsisldaagaaqaamaaqafabaWaaeWaceaafaqabeGabaaabaGaeiiFaWNaemOray0aaSbaaSqaaiabdUgaRbqabaGccqGHPiYXcuWGhbWrgaqeamaaBaaaleaacqWGQbGAaeqaaOGaeiiFaWhabaGaeGOmaidaaaGaayjkaiaawMcaaaWcbaGaem4AaSMaeyiyIKRaemyAaKgabeqdcqGHris5aaWcbaGaemysaKKaemysaKeakiaawIJ=aiabgkHiTmaayaaabaWaaabuaeaadaqadiqaauaabeqaceaaaeaacqGG8baFcuWGgbGrgaqeamaaBaaaleaacqWGPbqAaeqaaOGaeyykICSaem4raC0aaSbaaSqaaiabdYgaSbqabaGccqGG8baFaeaacqaIYaGmaaaacaGLOaGaayzkaaaaleaacqWGSbaBcqGHGjsUcqWGQbGAaeqaniabggHiLdaaleaacqWGjbqscqWGjbqscqWGjbqsaOGaayjo+dGaey4kaSYaaGbaaeaadaaeqbqaamaaqafabaWaaeWaceaafaqabeGabaaabaGaeiiFaWNaemOray0aaSbaaSqaaiabdUgaRbqabaGccqGHPiYXcqWGhbWrdaWgaaWcbaGaemiBaWgabeaakiabcYha8bqaaiabikdaYaaaaiaawIcacaGLPaaaaSqaaiabdYgaSjabgcMi5kabdQgaQbqab0GaeyyeIuoaaSqaaiabdUgaRjabgcMi5kabdMgaPbqab0GaeyyeIuoaaSqaaiabdMeajjabdAfawbGccaGL44pacqGH9aqpaeaadaagaaqaamaabmGabaqbaeqabiqaaaqaaiabcYha8jqbdAeagzaaraWaaSbaaSqaaiabdMgaPbqabaGccqGHPiYXcuWGhbWrgaqeamaaBaaaleaacqWGQbGAaeqaaOGaeiiFaWhabaGaeGOmaidaaaGaayjkaiaawMcaaaWcbaGaemysaKeakiaawIJ=aiabgkHiTmaayaaabaWaaabuaeaadaqadiqaauaabeqaceaaaeaacqGG8baFcqWGgbGrdaWgaaWcbaGaem4AaSgabeaakiabgMIihlqbdEeahzaaraWaaSbaaSqaaiabdQgaQbqabaGccqGG8baFaeaacqaIYaGmaaaacaGLOaGaayzkaaGaey4kaSYaaeWaceaafaqabeGabaaabaGaeiiFaWNaemOray0aaSbaaSqaaiabdMgaPbqabaGccqGHPiYXcuWGhbWrgaqeamaaBaaaleaacqWGQbGAaeqaaOGaeiiFaWhabaGaeGOmaidaaaGaayjkaiaawMcaaaWcbaGaem4AaSgabeqdcqGHris5aaWcbaGaemysaKKaemysaKeakiaawIJ=aiabgkHiTmaayaaabaWaaabuaeaadaqadiqaauaabeqaceaaaeaacqGG8baFcuWGgbGrgaqeamaaBaaaleaacqWGPbqAaeqaaOGaeyykICSaem4raC0aaSbaaSqaaiabdYgaSbqabaGccqGG8baFaeaacqaIYaGmaaaacaGLOaGaayzkaaGaey4kaSYaaeWaceaafaqabeGabaaabaGaeiiFaWNafmOrayKbaebadaWgaaWcbaGaemyAaKgabeaakiabgMIihlabdEeahnaaBaaaleaacqWGQbGAaeqaaOGaeiiFaWhabaGaeGOmaidaaaGaayjkaiaawMcaaaWcbaGaemiBaWgabeqdcqGHris5aaWcbaGaemysaKKaemysaKKaemysaKeakiaawIJ=aaqaaiabgUcaRmaayaaabaWaaabuaeaadaaeqbqaamaabmGabaqbaeqabiqaaaqaaiabcYha8jabdAeagnaaBaaaleaacqWGRbWAaeqaaOGaeyykICSaem4raC0aaSbaaSqaaiabdYgaSbqabaGccqGG8baFaeaacqaIYaGmaaaacaGLOaGaayzkaaGaeyOeI0caleaacqWGSbaBaeqaniabggHiLdaaleaacqWGRbWAaeqaniabggHiLdGcdaaeqbqaamaabmGabaqbaeqabiqaaaqaaiabcYha8jabdAeagnaaBaaaleaacqWGRbWAaeqaaOGaeyykICSaem4raC0aaSbaaSqaaiabdQgaQbqabaGccqGG8baFaeaacqaIYaGmaaaacaGLOaGaayzkaaGaeyOeI0caleaacqWGRbWAaeqaniabggHiLdGcdaaeqbqaamaabmGabaqbaeqabiqaaaqaaiabcYha8jabdAeagnaaBaaaleaacqWGPbqAaeqaaOGaeyykICSaem4raC0aaSbaaSqaaiabdYgaSbqabaGccqGG8baFaeaacqaIYaGmaaaacaGLOaGaayzkaaGaey4kaSYaaeWaceaafaqabeGabaaabaGaeiiFaWNaemOray0aaSbaaSqaaiabdMgaPbqabaGccqGHPiYXcqWGhbWrdaWgaaWcbaGaemOAaOgabeaakiabcYha8bqaaiabikdaYaaaaiaawIcacaGLPaaaaSqaaiabdYgaSbqab0GaeyyeIuoaaSqaaiabdMeajjabdAfawbGccaGL44paaaGaaCzcaiaaxMaadaqadiqaaiabisda0aGaayjkaiaawMcaaaaa@3050@

Let

Sj=∑k(|Fk∩Gj|2)S′i=∑l(|Fi∩Gl|2)S¯j=∑k(|Fk∩G¯j|2)S¯′i=∑l(|F¯i∩Gl|2)S=∑k∑l(|Fk∩Gl|2)     (5)
 MathType@MTEF@5@5@+=feaafiart1ev1aaatCvAUfKttLearuWrP9MDH5MBPbIqV92AaeXatLxBI9gBaebbnrfifHhDYfgasaacH8akY=wiFfYdH8Gipec8Eeeu0xXdbba9frFj0=OqFfea0dXdd9vqai=hGuQ8kuc9pgc9s8qqaq=dirpe0xb9q8qiLsFr0=vr0=vr0dc8meaabaqaciaacaGaaeqabaqabeGadaaakeaafaqadeqbbaaaaeaacqWGtbWudaWgaaWcbaGaemOAaOgabeaakiabg2da9maaqafabaWaaeWaceaafaqabeGabaaabaGaeiiFaWNaemOray0aaSbaaSqaaiabdUgaRbqabaGccqGHPiYXcqWGhbWrdaWgaaWcbaGaemOAaOgabeaakiabcYha8bqaaiabikdaYaaaaiaawIcacaGLPaaaaSqaaiabdUgaRbqab0GaeyyeIuoaaOqaaiqbdofatzaafaWaaSbaaSqaaiabdMgaPbqabaGccqGH9aqpdaaeqbqaamaabmGabaqbaeqabiqaaaqaaiabcYha8jabdAeagnaaBaaaleaacqWGPbqAaeqaaOGaeyykICSaem4raC0aaSbaaSqaaiabdYgaSbqabaGccqGG8baFaeaacqaIYaGmaaaacaGLOaGaayzkaaaaleaacqWGSbaBaeqaniabggHiLdaakeaacuWGtbWugaqeamaaBaaaleaacqWGQbGAaeqaaOGaeyypa0ZaaabuaeaadaqadiqaauaabeqaceaaaeaacqGG8baFcqWGgbGrdaWgaaWcbaGaem4AaSgabeaakiabgMIihlqbdEeahzaaraWaaSbaaSqaaiabdQgaQbqabaGccqGG8baFaeaacqaIYaGmaaaacaGLOaGaayzkaaaaleaacqWGRbWAaeqaniabggHiLdaakeaacuWGtbWugaqegaqbamaaBaaaleaacqWGPbqAaeqaaOGaeyypa0ZaaabuaeaadaqadiqaauaabeqaceaaaeaacqGG8baFcuWGgbGrgaqeamaaBaaaleaacqWGPbqAaeqaaOGaeyykICSaem4raC0aaSbaaSqaaiabdYgaSbqabaGccqGG8baFaeaacqaIYaGmaaaacaGLOaGaayzkaaaaleaacqWGSbaBaeqaniabggHiLdaakeaacqWGtbWucqGH9aqpdaaeqbqaamaaqafabaWaaeWaceaafaqabeGabaaabaGaeiiFaWNaemOray0aaSbaaSqaaiabdUgaRbqabaGccqGHPiYXcqWGhbWrdaWgaaWcbaGaemiBaWgabeaakiabcYha8bqaaiabikdaYaaaaiaawIcacaGLPaaaaSqaaiabdYgaSbqab0GaeyyeIuoaaSqaaiabdUgaRbqab0GaeyyeIuoaaaGccaWLjaGaaCzcamaabmGabaGaeGynaudacaGLOaGaayzkaaaaaa@9604@

We can ignore leaf subtrees, so we need to compute *id*_*v' *_different S¯
 MathType@MTEF@5@5@+=feaafiart1ev1aaatCvAUfKttLearuWrP9MDH5MBPbIqV92AaeXatLxBI9gBaebbnrfifHhDYfgasaacH8akY=wiFfYdH8Gipec8Eeeu0xXdbba9frFj0=OqFfea0dXdd9vqai=hGuQ8kuc9pgc9s8qqaq=dirpe0xb9q8qiLsFr0=vr0=vr0dc8meaabaqaciaacaGaaeqabaqabeGadaaakeaacuWGtbWugaqeaaaa@2DF3@_*j*_'s and *S*_*j*_'s which can each be computed in *O*(*id*_*v*_) time. Symmetrically each of the *id*_*v *_S¯′i
 MathType@MTEF@5@5@+=feaafiart1ev1aaatCvAUfKttLearuWrP9MDH5MBPbIqV92AaeXatLxBI9gBaebbnrfifHhDYfgasaacH8akY=wiFfYdH8Gipec8Eeeu0xXdbba9frFj0=OqFfea0dXdd9vqai=hGuQ8kuc9pgc9s8qqaq=dirpe0xb9q8qiLsFr0=vr0=vr0dc8meaabaqaciaacaGaaeqabaqabeGadaaakeaacuWGtbWugaqegaqbamaaBaaaleaacqWGPbqAaeqaaaaa@2F85@'s and S′i
 MathType@MTEF@5@5@+=feaafiart1ev1aaatCvAUfKttLearuWrP9MDH5MBPbIqV92AaeXatLxBI9gBaebbnrfifHhDYfgasaacH8akY=wiFfYdH8Gipec8Eeeu0xXdbba9frFj0=OqFfea0dXdd9vqai=hGuQ8kuc9pgc9s8qqaq=dirpe0xb9q8qiLsFr0=vr0=vr0dc8meaabaqaciaacaGaaeqabaqabeGadaaakeaacuWGtbWugaqbamaaBaaaleaacqWGPbqAaeqaaaaa@2F6E@'s takes time *O*(id′v
 MathType@MTEF@5@5@+=feaafiart1ev1aaatCvAUfKttLearuWrP9MDH5MBPbIqV92AaeXatLxBI9gBaebbnrfifHhDYfgasaacH8akY=wiFfYdH8Gipec8Eeeu0xXdbba9frFj0=OqFfea0dXdd9vqai=hGuQ8kuc9pgc9s8qqaq=dirpe0xb9q8qiLsFr0=vr0=vr0dc8meaabaqaciaacaGaaeqabaqabeGadaaakeaacqWGPbqAcuWGKbazgaqbamaaBaaaleaacqWG2bGDaeqaaaaa@3105@) to compute, and the total time of computing *S *is *O*(*id*_*v*_*id*_*v'*_). The total time of computing all sums mentioned is thus *O*(*id*_*v*_*id*_*v'*_) and this is the key to reducing the time usage of shared(*v*,*v'*). Using the sums we can express (4) as:

(|F¯i∩G¯j|2)︸I−S¯j+(|Fi∩G¯j|2)︸II−S¯′i+(|F¯i∩Gj|2)︸III+S−Sj−S′i+(|Fi∩Gj|2)︸IV
 MathType@MTEF@5@5@+=feaafiart1ev1aaatCvAUfKttLearuWrP9MDH5MBPbIqV92AaeXatLxBI9gBaebbnrfifHhDYfgasaacH8akY=wiFfYdH8Gipec8Eeeu0xXdbba9frFj0=OqFfea0dXdd9vqai=hGuQ8kuc9pgc9s8qqaq=dirpe0xb9q8qiLsFr0=vr0=vr0dc8meaabaqaciaacaGaaeqabaqabeGadaaakeaadaagaaqaamaabmGabaqbaeqabiqaaaqaaiabcYha8jqbdAeagzaaraWaaSbaaSqaaiabdMgaPbqabaGccqGHPiYXcuWGhbWrgaqeamaaBaaaleaacqWGQbGAaeqaaOGaeiiFaWhabaGaeGOmaidaaaGaayjkaiaawMcaaaWcbaGaemysaKeakiaawIJ=aiabgkHiTmaayaaabaGafm4uamLbaebadaWgaaWcbaGaemOAaOgabeaakiabgUcaRmaabmGabaqbaeqabiqaaaqaaiabcYha8jabdAeagnaaBaaaleaacqWGPbqAaeqaaOGaeyykICSafm4raCKbaebadaWgaaWcbaGaemOAaOgabeaakiabcYha8bqaaiabikdaYaaaaiaawIcacaGLPaaaaSqaaiabdMeajjabdMeajbGccaGL44pacqGHsisldaagaaqaaiqbdofatzaaryaafaWaaSbaaSqaaiabdMgaPbqabaGccqGHRaWkdaqadiqaauaabeqaceaaaeaacqGG8baFcuWGgbGrgaqeamaaBaaaleaacqWGPbqAaeqaaOGaeyykICSaem4raC0aaSbaaSqaaiabdQgaQbqabaGccqGG8baFaeaacqaIYaGmaaaacaGLOaGaayzkaaaaleaacqWGjbqscqWGjbqscqWGjbqsaOGaayjo+dGaey4kaSYaaGbaaeaacqWGtbWucqGHsislcqWGtbWudaWgaaWcbaGaemOAaOgabeaakiabgkHiTiqbdofatzaafaWaaSbaaSqaaiabdMgaPbqabaGccqGHRaWkdaqadiqaauaabeqaceaaaeaacqGG8baFcqWGgbGrdaWgaaWcbaGaemyAaKgabeaakiabgMIihlabdEeahnaaBaaaleaacqWGQbGAaeqaaOGaeiiFaWhabaGaeGOmaidaaaGaayjkaiaawMcaaaWcbaGaemysaKKaemOvayfakiaawIJ=aaaa@8394@

Provided that the sums S¯
 MathType@MTEF@5@5@+=feaafiart1ev1aaatCvAUfKttLearuWrP9MDH5MBPbIqV92AaeXatLxBI9gBaebbnrfifHhDYfgasaacH8akY=wiFfYdH8Gipec8Eeeu0xXdbba9frFj0=OqFfea0dXdd9vqai=hGuQ8kuc9pgc9s8qqaq=dirpe0xb9q8qiLsFr0=vr0=vr0dc8meaabaqaciaacaGaaeqabaqabeGadaaakeaacuWGtbWugaqeaaaa@2DF3@_*j*_, S¯′i
 MathType@MTEF@5@5@+=feaafiart1ev1aaatCvAUfKttLearuWrP9MDH5MBPbIqV92AaeXatLxBI9gBaebbnrfifHhDYfgasaacH8akY=wiFfYdH8Gipec8Eeeu0xXdbba9frFj0=OqFfea0dXdd9vqai=hGuQ8kuc9pgc9s8qqaq=dirpe0xb9q8qiLsFr0=vr0=vr0dc8meaabaqaciaacaGaaeqabaqabeGadaaakeaacuWGtbWugaqegaqbamaaBaaaleaacqWGPbqAaeqaaaaa@2F85@, *S*_*j*_, S′i
 MathType@MTEF@5@5@+=feaafiart1ev1aaatCvAUfKttLearuWrP9MDH5MBPbIqV92AaeXatLxBI9gBaebbnrfifHhDYfgasaacH8akY=wiFfYdH8Gipec8Eeeu0xXdbba9frFj0=OqFfea0dXdd9vqai=hGuQ8kuc9pgc9s8qqaq=dirpe0xb9q8qiLsFr0=vr0=vr0dc8meaabaqaciaacaGaaeqabaqabeGadaaakeaacuWGtbWugaqbamaaBaaaleaacqWGPbqAaeqaaaaa@2F6E@ and *S *have been calculated, (4) can be calculated in time *O*(l). Since calculation of the sums is independent on the calculation of shared(*v*, *v'*), these calculations can be done serially as shown in the algorithm below, thereby reducing the time usage of shared(*T*, *T'*) to:

∑v∈T∑v′∈T′idvidv′=∑v∈Tidv∑v′∈T′idv′=2(|V|−1)⋅2(|V′|−1)=O(|V||V′|)
 MathType@MTEF@5@5@+=feaafiart1ev1aaatCvAUfKttLearuWrP9MDH5MBPbIqV92AaeXatLxBI9gBaebbnrfifHhDYfgasaacH8akY=wiFfYdH8Gipec8Eeeu0xXdbba9frFj0=OqFfea0dXdd9vqai=hGuQ8kuc9pgc9s8qqaq=dirpe0xb9q8qiLsFr0=vr0=vr0dc8meaabaqaciaacaGaaeqabaqabeGadaaakeaadaaeqbqaamaaqafabaGaemyAaKMaemizaq2aaSbaaSqaaiabdAha2bqabaGccqWGPbqAcqWGKbazdaWgaaWcbaGafmODayNbauaaaeqaaaqaaiqbdAha2zaafaGaeyicI4SafmivaqLbauaaaeqaniabggHiLdGccqGH9aqpdaaeqbqaaiabdMgaPjabdsgaKnaaBaaaleaacqWG2bGDaeqaaaqaaiabdAha2jabgIGiolabdsfaubqab0GaeyyeIuoakmaaqafabaGaemyAaKMaemizaq2aaSbaaSqaaiqbdAha2zaafaaabeaaaeaacuWG2bGDgaqbaiabgIGiolqbdsfauzaafaaabeqdcqGHris5aOGaeyypa0JaeGOmaiZaaeWaceaacqGG8baFcqWGwbGvcqGG8baFcqGHsislcqaIXaqmaiaawIcacaGLPaaacqGHflY1cqaIYaGmdaqadiqaaiabcYha8jqbdAfawzaafaGaeiiFaWNaeyOeI0IaeGymaedacaGLOaGaayzkaaGaeyypa0Jaem4ta80aaeWaceaacqGG8baFcqWGwbGvcqGG8baFcqGG8baFcuWGwbGvgaqbaiabcYha8bGaayjkaiaawMcaaaWcbaGaemODayNaeyicI4SaemivaqfabeqdcqGHris5aaaa@7911@

ALGORITHM – CALCULATING THE NUMBER OF SHARED BUTTERFLY QUARTETS BETWEEN *T *AND *T'*

**Requires: ***T*, *T' *two input trees with the same leaf set.

**Ensures: ***Res *= shared(*T*, *T'*)

*Res *← 0

**for ***v *internal node in *T ***do**

   **for ***v' *internal node in *T' ***do**

      Calculate sums S¯
 MathType@MTEF@5@5@+=feaafiart1ev1aaatCvAUfKttLearuWrP9MDH5MBPbIqV92AaeXatLxBI9gBaebbnrfifHhDYfgasaacH8akY=wiFfYdH8Gipec8Eeeu0xXdbba9frFj0=OqFfea0dXdd9vqai=hGuQ8kuc9pgc9s8qqaq=dirpe0xb9q8qiLsFr0=vr0=vr0dc8meaabaqaciaacaGaaeqabaqabeGadaaakeaacuWGtbWugaqeaaaa@2DF3@_*j*_, S¯′i
 MathType@MTEF@5@5@+=feaafiart1ev1aaatCvAUfKttLearuWrP9MDH5MBPbIqV92AaeXatLxBI9gBaebbnrfifHhDYfgasaacH8akY=wiFfYdH8Gipec8Eeeu0xXdbba9frFj0=OqFfea0dXdd9vqai=hGuQ8kuc9pgc9s8qqaq=dirpe0xb9q8qiLsFr0=vr0=vr0dc8meaabaqaciaacaGaaeqabaqabeGadaaakeaacuWGtbWugaqegaqbamaaBaaaleaacqWGPbqAaeqaaaaa@2F85@, *S*_*j*_, S′i
 MathType@MTEF@5@5@+=feaafiart1ev1aaatCvAUfKttLearuWrP9MDH5MBPbIqV92AaeXatLxBI9gBaebbnrfifHhDYfgasaacH8akY=wiFfYdH8Gipec8Eeeu0xXdbba9frFj0=OqFfea0dXdd9vqai=hGuQ8kuc9pgc9s8qqaq=dirpe0xb9q8qiLsFr0=vr0=vr0dc8meaabaqaciaacaGaaeqabaqabeGadaaakeaacuWGtbWugaqbamaaBaaaleaacqWGPbqAaeqaaaaa@2F6E@ and *S*

      *Res *← *Res *+ shared(*v*, *v'*)

   **end for**

end for

*Res *← Res2
 MathType@MTEF@5@5@+=feaafiart1ev1aaatCvAUfKttLearuWrP9MDH5MBPbIqV92AaeXatLxBI9gBaebbnrfifHhDYfgasaacH8akY=wiFfYdH8Gipec8Eeeu0xXdbba9frFj0=OqFfea0dXdd9vqai=hGuQ8kuc9pgc9s8qqaq=dirpe0xb9q8qiLsFr0=vr0=vr0dc8meaabaqaciaacaGaaeqabaqabeGadaaakeaadaWcaaqaaGqaciab=jfasjab=vgaLjab=nhaZbqaaiabikdaYaaaaaa@319C@

### Counting nonshared butterfly quartets

**Figure 9:**
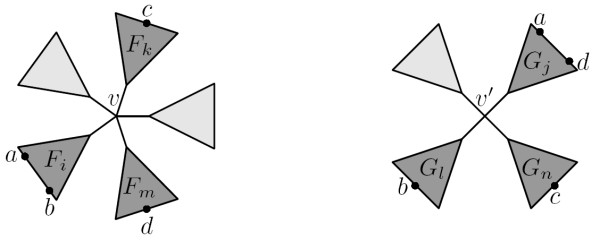
Internal nodes *v *∈ *T *claiming the quartet *ab*|*cd *and *v' *∈ *T' *claiming the quartet *ad*|*bc*.

For each pair of internal nodes *v*, *v' *we want to count the number of nonshared butterfly quartets claimed by both internal nodes, nonshared(*v*, *v'*). Such quartets have the property that a pair of leaves found in the same subtree of *v *will be found in different subtrees of *v' *and vice versa, i.e. a nonshared quartet with leaves *a*, *b*, *c *and *d*, has a ∈ *F*_*i *_∩ *G*_*j*_, *b *∈ *F*_*i *_∩ *G*_*l*_, *c *∈ *F*_*k *_∩ *G*_*n *_and *d *∈ *F*_*m *_∩ *G*_*j *_(see Fig. 9). The following expression counts all nonshared quartets related to a pair of nodes *v *and *v'*, obeying that if two leaves of the quartet are in one subtree of *v *they are in different subtrees of *v' *and vice versa:

∑i∑j|Fi∩Gj||Fi∩G¯j||F¯i∩G¯j||F¯i∩Gj|     (6)
 MathType@MTEF@5@5@+=feaafiart1ev1aaatCvAUfKttLearuWrP9MDH5MBPbIqV92AaeXatLxBI9gBaebbnrfifHhDYfgasaacH8akY=wiFfYdH8Gipec8Eeeu0xXdbba9frFj0=OqFfea0dXdd9vqai=hGuQ8kuc9pgc9s8qqaq=dirpe0xb9q8qiLsFr0=vr0=vr0dc8meaabaqaciaacaGaaeqabaqabeGadaaakeaadaaeqbqaamaaqafabaGaeiiFaWNaemOray0aaSbaaSqaaiabdMgaPbqabaGccqGHPiYXcqWGhbWrdaWgaaWcbaGaemOAaOgabeaakiabcYha8jabcYha8jabdAeagnaaBaaaleaacqWGPbqAaeqaaOGaeyykICSafm4raCKbaebadaWgaaWcbaGaemOAaOgabeaakiabcYha8jabcYha8jqbdAeagzaaraWaaSbaaSqaaiabdMgaPbqabaGccqGHPiYXcuWGhbWrgaqeamaaBaaaleaacqWGQbGAaeqaaOGaeiiFaWNaeiiFaWNafmOrayKbaebadaWgaaWcbaGaemyAaKgabeaakiabgMIihlabdEeahnaaBaaaleaacqWGQbGAaeqaaOGaeiiFaWhaleaacqWGQbGAaeqaniabggHiLdaaleaacqWGPbqAaeqaniabggHiLdGccaWLjaGaaCzcamaabmGabaGaeGOnaydacaGLOaGaayzkaaaaaa@5F95@

Even though (6) satisfies the property of nonshared quartets, it possibly counts more than the number of nonshared quartets claimed by an internal node in each tree. The problem is that given two internal nodes, they do not nescessarily *claim *the quartets counted by (6). If we denote the leaves of an nonshared quartet *a*, *b*, *c *and *d*, the first, second, third and fourth factors in (6) counts the number of choices of *a*, *b*, *c *and *d *respectively. The first and second factor choose *a *and *b *from *F*_*i*_, while the third and fourth choose *c *and *d *from F¯
 MathType@MTEF@5@5@+=feaafiart1ev1aaatCvAUfKttLearuWrP9MDH5MBPbIqV92AaeXatLxBI9gBaebbnrfifHhDYfgasaacH8akY=wiFfYdH8Gipec8Eeeu0xXdbba9frFj0=OqFfea0dXdd9vqai=hGuQ8kuc9pgc9s8qqaq=dirpe0xb9q8qiLsFr0=vr0=vr0dc8meaabaqaciaacaGaaeqabaqabeGadaaakeaacuWGgbGrgaqeaaaa@2DD9@_*i*_. In the cases where *c *and *d *are chosen from the same subtree *F*_*k*_, *k *≠ *i *of *v*, *v *does not claim the quartet. We must subtract these quartets, which can be counted as:

∑i∑j∑k≠i|Fi∩Gj||Fi∩G¯j||Fk∩G¯j||Fk∩Gj|     (7)
 MathType@MTEF@5@5@+=feaafiart1ev1aaatCvAUfKttLearuWrP9MDH5MBPbIqV92AaeXatLxBI9gBaebbnrfifHhDYfgasaacH8akY=wiFfYdH8Gipec8Eeeu0xXdbba9frFj0=OqFfea0dXdd9vqai=hGuQ8kuc9pgc9s8qqaq=dirpe0xb9q8qiLsFr0=vr0=vr0dc8meaabaqaciaacaGaaeqabaqabeGadaaakeaadaaeqbqaamaaqafabaWaaabuaeaacqGG8baFcqWGgbGrdaWgaaWcbaGaemyAaKgabeaakiabgMIihlabdEeahnaaBaaaleaacqWGQbGAaeqaaOGaeiiFaWNaeiiFaWNaemOray0aaSbaaSqaaiabdMgaPbqabaGccqGHPiYXcuWGhbWrgaqeamaaBaaaleaacqWGQbGAaeqaaOGaeiiFaWNaeiiFaWNaemOray0aaSbaaSqaaiabdUgaRbqabaGccqGHPiYXcuWGhbWrgaqeamaaBaaaleaacqWGQbGAaeqaaOGaeiiFaWNaeiiFaWNaemOray0aaSbaaSqaaiabdUgaRbqabaGccqGHPiYXcqWGhbWrdaWgaaWcbaGaemOAaOgabeaakiabcYha8bWcbaGaem4AaSMaeyiyIKRaemyAaKgabeqdcqGHris5aaWcbaGaemOAaOgabeqdcqGHris5aOGaaCzcaiaaxMaadaqadiqaaiabiEda3aGaayjkaiaawMcaaaWcbaGaemyAaKgabeqdcqGHris5aaaa@6613@

Similarly there are cases where *b *and *c *are chosen from the same subtree *G*_*l*_, *l *≠ *j *of *v'*, which we must also subtract. These can be counted as:

∑i∑j∑l≠j|Fi∩Gj||Fi∩Gl||F¯i∩Gl||F¯i∩Gj|     (8)
 MathType@MTEF@5@5@+=feaafiart1ev1aaatCvAUfKttLearuWrP9MDH5MBPbIqV92AaeXatLxBI9gBaebbnrfifHhDYfgasaacH8akY=wiFfYdH8Gipec8Eeeu0xXdbba9frFj0=OqFfea0dXdd9vqai=hGuQ8kuc9pgc9s8qqaq=dirpe0xb9q8qiLsFr0=vr0=vr0dc8meaabaqaciaacaGaaeqabaqabeGadaaakeaadaaeqbqaamaaqafabaWaaabuaeaacqGG8baFcqWGgbGrdaWgaaWcbaGaemyAaKgabeaakiabgMIihlabdEeahnaaBaaaleaacqWGQbGAaeqaaOGaeiiFaWNaeiiFaWNaemOray0aaSbaaSqaaiabdMgaPbqabaGccqGHPiYXcqWGhbWrdaWgaaWcbaGaemiBaWgabeaakiabcYha8jabcYha8jqbdAeagzaaraWaaSbaaSqaaiabdMgaPbqabaGccqGHPiYXcqWGhbWrdaWgaaWcbaGaemiBaWgabeaakiabcYha8jabcYha8jqbdAeagzaaraWaaSbaaSqaaiabdMgaPbqabaGccqGHPiYXcqWGhbWrdaWgaaWcbaGaemOAaOgabeaakiabcYha8bWcbaGaemiBaWMaeyiyIKRaemOAaOgabeqdcqGHris5aaWcbaGaemOAaOgabeqdcqGHris5aaWcbaGaemyAaKgabeqdcqGHris5aOGaaCzcaiaaxMaadaqadiqaaiabiIda4aGaayjkaiaawMcaaaaa@6619@

The cases where both *c *and *d *are chosen from the same subtree *F*_*k*_, *k *≠ *i *of *v *and *b *and *c *are chosen from the same subtree *G*_*l*_, *l *≠ *j *of *v' *are included in both the expressions above and therefore they must be added again. The following expression counts the number of these cases:

∑i∑j∑k≠i∑l≠j|Fi∩Gj||Fi∩Gl||Fk∩Gl||Fk∩Gj|     (9)
 MathType@MTEF@5@5@+=feaafiart1ev1aaatCvAUfKttLearuWrP9MDH5MBPbIqV92AaeXatLxBI9gBaebbnrfifHhDYfgasaacH8akY=wiFfYdH8Gipec8Eeeu0xXdbba9frFj0=OqFfea0dXdd9vqai=hGuQ8kuc9pgc9s8qqaq=dirpe0xb9q8qiLsFr0=vr0=vr0dc8meaabaqaciaacaGaaeqabaqabeGadaaakeaadaaeqbqaamaaqafabaWaaabuaeaadaaeqbqaaiabcYha8jabdAeagnaaBaaaleaacqWGPbqAaeqaaOGaeyykICSaem4raC0aaSbaaSqaaiabdQgaQbqabaGccqGG8baFcqGG8baFcqWGgbGrdaWgaaWcbaGaemyAaKgabeaakiabgMIihlabdEeahnaaBaaaleaacqWGSbaBaeqaaOGaeiiFaWNaeiiFaWNaemOray0aaSbaaSqaaiabdUgaRbqabaGccqGHPiYXcqWGhbWrdaWgaaWcbaGaemiBaWgabeaakiabcYha8jabcYha8jabdAeagnaaBaaaleaacqWGRbWAaeqaaOGaeyykICSaem4raC0aaSbaaSqaaiabdQgaQbqabaGccqGG8baFaSqaaiabdYgaSjabgcMi5kabdQgaQbqab0GaeyyeIuoaaSqaaiabdUgaRjabgcMi5kabdMgaPbqab0GaeyyeIuoaaSqaaiabdQgaQbqab0GaeyyeIuoaaSqaaiabdMgaPbqab0GaeyyeIuoakiaaxMaacaWLjaWaaeWaceaacqaI5aqoaiaawIcacaGLPaaaaaa@6C97@

Combining equations (6), (7), (8) and (9), gives a way of calculating the number of nonshared quartets between two internal nodes *v *and *v'*:

nonshared(v,v′)=∑i∑j(|Fi∩Gj||Fi∩G¯j||F¯i∩G¯j||F¯i∩Gj|−∑k≠i|Fi∩Gj||Fi∩G¯j||Fk∩G¯j||Fk∩Gj|−∑l≠j|Fi∩Gj||Fi∩Gl||F¯i∩Gl||F¯i∩Gj|+∑k≠i∑l≠j|Fi∩Gj||Fi∩Gl||Fk∩Gl||Fk∩Gj|)     (10)
 MathType@MTEF@5@5@+=feaafiart1ev1aaatCvAUfKttLearuWrP9MDH5MBPbIqV92AaeXatLxBI9gBaebbnrfifHhDYfgasaacH8akY=wiFfYdH8Gipec8Eeeu0xXdbba9frFj0=OqFfea0dXdd9vqai=hGuQ8kuc9pgc9s8qqaq=dirpe0xb9q8qiLsFr0=vr0=vr0dc8meaabaqaciaacaGaaeqabaqabeGadaaakeaafaqadeabbaaaaeaaieaacqWFUbGBcqWFVbWBcqWFUbGBcqWFZbWCcqWFObaAcqWFHbqycqWFYbGCcqWFLbqzcqWFKbazdaqadiqaaGqaciab+zha2jab+XcaSiqb+zha2zaafaaacaGLOaGaayzkaaGaeyypa0ZaaabuaeaadaaeqbqaamaabeGabaGaeiiFaWNaemOray0aaSbaaSqaaiabdMgaPbqabaGccqGHPiYXcqWGhbWrdaWgaaWcbaGaemOAaOgabeaakiabcYha8jabcYha8jabdAeagnaaBaaaleaacqWGPbqAaeqaaOGaeyykICSafm4raCKbaebadaWgaaWcbaGaemOAaOgabeaakiabcYha8jabcYha8jqbdAeagzaaraWaaSbaaSqaaiabdMgaPbqabaGccqGHPiYXcuWGhbWrgaqeamaaBaaaleaacqWGQbGAaeqaaOGaeiiFaWNaeiiFaWNafmOrayKbaebadaWgaaWcbaGaemyAaKgabeaakiabgMIihlabdEeahnaaBaaaleaacqWGQbGAaeqaaOGaeiiFaWhacaGLOaaaaSqaaiabdQgaQbqab0GaeyyeIuoaaSqaaiabdMgaPbqab0GaeyyeIuoaaOqaceaawpGaaCzcaiabgkHiTmaaqafabaGaeiiFaWNaemOray0aaSbaaSqaaiabdMgaPbqabaGccqGHPiYXcqWGhbWrdaWgaaWcbaGaemOAaOgabeaakiabcYha8jabcYha8jabdAeagnaaBaaaleaacqWGPbqAaeqaaOGaeyykICSafm4raCKbaebadaWgaaWcbaGaemOAaOgabeaakiabcYha8jabcYha8jabdAeagnaaBaaaleaacqWGRbWAaeqaaOGaeyykICSafm4raCKbaebadaWgaaWcbaGaemOAaOgabeaakiabcYha8jabcYha8jabdAeagnaaBaaaleaacqWGRbWAaeqaaOGaeyykICSaem4raC0aaSbaaSqaaiabdQgaQbqabaGccqGG8baFaSqaaiabdUgaRjabgcMi5kabdMgaPbqab0GaeyyeIuoaaOqaciaakpaa6pGaaCzcaiabgkHiTmaaqafabaGaeiiFaWNaemOray0aaSbaaSqaaiabdMgaPbqabaGccqGHPiYXcqWGhbWrdaWgaaWcbaGaemOAaOgabeaakiabcYha8jabcYha8jabdAeagnaaBaaaleaacqWGPbqAaeqaaOGaeyykICSaem4raC0aaSbaaSqaaiabdYgaSbqabaGccqGG8baFcqGG8baFcuWGgbGrgaqeamaaBaaaleaacqWGPbqAaeqaaOGaeyykICSaem4raC0aaSbaaSqaaiabdYgaSbqabaGccqGG8baFcqGG8baFcuWGgbGrgaqeamaaBaaaleaacqWGPbqAaeqaaOGaeyykICSaem4raC0aaSbaaSqaaiabdQgaQbqabaGccqGG8baFaSqaaiabdYgaSjabgcMi5kabdQgaQbqab0GaeyyeIuoaaOqaamaabiGabiWaaO5aaibbaihbcaWLjaGaey4kaSYaaabuaeaadaaeqbqaaiabcYha8jabdAeagnaaBaaaleaacqWGPbqAaeqaaOGaeyykICSaem4raC0aaSbaaSqaaiabdQgaQbqabaGccqGG8baFcqGG8baFcqWGgbGrdaWgaaWcbaGaemyAaKgabeaakiabgMIihlabdEeahnaaBaaaleaacqWGSbaBaeqaaOGaeiiFaWNaeiiFaWNaemOray0aaSbaaSqaaiabdUgaRbqabaGccqGHPiYXcqWGhbWrdaWgaaWcbaGaemiBaWgabeaakiabcYha8jabcYha8jabdAeagnaaBaaaleaacqWGRbWAaeqaaOGaeyykICSaem4raC0aaSbaaSqaaiabdQgaQbqabaGccqGG8baFaSqaaiabdYgaSjabgcMi5kabdQgaQbqab0GaeyyeIuoaaSqaaiabdUgaRjabgcMi5kabdMgaPbqab0GaeyyeIuoaaOGaayzkaaaaaiaaxMaacaWLjaWaaeWaceaacqaIXaqmcqaIWaamaiaawIcacaGLPaaaaaa@0F94@

Assuming that the trees have a nonshared quartet with topology form *ab*|*cd *in *T*, and *ad*|*bc *in *T'*, there are two internal nodes in each tree claiming the quartet: *v*_*ab*|*c*|*d *_and *v*_*cd*|*a*|*b *_in *T *and v′ad|b|c
 MathType@MTEF@5@5@+=feaafiart1ev1aaatCvAUfKttLearuWrP9MDH5MBPbIqV92AaeXatLxBI9gBaebbnrfifHhDYfgasaacH8akY=wiFfYdH8Gipec8Eeeu0xXdbba9frFj0=OqFfea0dXdd9vqai=hGuQ8kuc9pgc9s8qqaq=dirpe0xb9q8qiLsFr0=vr0=vr0dc8meaabaqaciaacaGaaeqabaqabeGadaaakeaacuWG2bGDgaqbamaaBaaaleaacqWGHbqycqWGKbazcqGG8baFcqWGIbGycqGG8baFcqWGJbWyaeqaaaaa@3691@ and v′bc|a|d
 MathType@MTEF@5@5@+=feaafiart1ev1aaatCvAUfKttLearuWrP9MDH5MBPbIqV92AaeXatLxBI9gBaebbnrfifHhDYfgasaacH8akY=wiFfYdH8Gipec8Eeeu0xXdbba9frFj0=OqFfea0dXdd9vqai=hGuQ8kuc9pgc9s8qqaq=dirpe0xb9q8qiLsFr0=vr0=vr0dc8meaabaqaciaacaGaaeqabaqabeGadaaakeaacuWG2bGDgaqbamaaBaaaleaacqWGIbGycqWGJbWycqGG8baFcqWGHbqycqGG8baFcqWGKbazaeqaaaaa@3691@ in *T'*. All of the four combinations of these will identify the quartet as nonshared. Therefore the total number of nonshared quartets between the two trees is:

nonshared(T,T′)=14∑v∈T∑v′∈T′nonshared(v,v′)
 MathType@MTEF@5@5@+=feaafiart1ev1aaatCvAUfKttLearuWrP9MDH5MBPbIqV92AaeXatLxBI9gBaebbnrfifHhDYfgasaacH8akY=wiFfYdH8Gipec8Eeeu0xXdbba9frFj0=OqFfea0dXdd9vqai=hGuQ8kuc9pgc9s8qqaq=dirpe0xb9q8qiLsFr0=vr0=vr0dc8meaabaqaciaacaGaaeqabaqabeGadaaakeaacqqGUbGBcqqGVbWBcqqGUbGBcqqGZbWCcqqGObaAcqqGHbqycqqGYbGCcqqGLbqzcqqGKbazcqGGOaakcqWGubavcqGGSaalcuWGubavgaqbaiabcMcaPiabg2da9maalaaabaGaeGymaedabaGaeGinaqdaamaaqafabaWaaabuaeaacqqGUbGBcqqGVbWBcqqGUbGBcqqGZbWCcqqGObaAcqqGHbqycqqGYbGCcqqGLbqzcqqGKbazcqGGOaakcqWG2bGDcqGGSaalcuWG2bGDgaqbaiabcMcaPaWcbaGafmODayNbauaacqGHiiIZcuWGubavgaqbaaqab0GaeyyeIuoaaSqaaiabdAha2jabgIGiolabdsfaubqab0GaeyyeIuoaaaa@5F68@

Direct computation of nonshared(*v*, *v'*) using (10) takes time *O*(dv2dv′2
 MathType@MTEF@5@5@+=feaafiart1ev1aaatCvAUfKttLearuWrP9MDH5MBPbIqV92AaeXatLxBI9gBaebbnrfifHhDYfgasaacH8akY=wiFfYdH8Gipec8Eeeu0xXdbba9frFj0=OqFfea0dXdd9vqai=hGuQ8kuc9pgc9s8qqaq=dirpe0xb9q8qiLsFr0=vr0=vr0dc8meaabaqaciaacaGaaeqabaqabeGadaaakeaacqWGKbazdaqhaaWcbaGaemODayhabaGaeGOmaidaaOGaemizaq2aa0baaSqaaiqbdAha2zaafaaabaGaeGOmaidaaaaa@348C@). Each of the subtrees has to be intersected with two disjoint sets in each of the sums. This means that if a subtree is only a leaf, at least one of these intersections will be zero, and the term will be zero. Therefore we can ignore subtrees that consist of a single leaf, just like when computing shared(*T*, *T'*), and reduce the time usage to *O*(idv2idv′2
 MathType@MTEF@5@5@+=feaafiart1ev1aaatCvAUfKttLearuWrP9MDH5MBPbIqV92AaeXatLxBI9gBaebbnrfifHhDYfgasaacH8akY=wiFfYdH8Gipec8Eeeu0xXdbba9frFj0=OqFfea0dXdd9vqai=hGuQ8kuc9pgc9s8qqaq=dirpe0xb9q8qiLsFr0=vr0=vr0dc8meaabaqaciaacaGaaeqabaqabeGadaaakeaacqWGPbqAcqWGKbazdaqhaaWcbaGaemODayhabaGaeGOmaidaaOGaemyAaKMaemizaq2aa0baaSqaaiqbdAha2zaafaaabaGaeGOmaidaaaaa@3742@). The time usage of the calculation of nonshared(*v*, *v'*) can be further improved by rewriting (9):

∑i∑j∑k≠i∑l≠j|Fi∩Gj||Fi∩Gl||Fk∩Gl||Fk∩Gj|=∑i∑j|Fi∩Gj|∑k≠i|Fk∩Gj|∑l≠j|Fi∩Gl||Fk∩Gl|=∑i∑j|Fi∩Gj|∑k≠i|Fk∩Gj|(∑l|Fi∩Gl||Fk∩Gl|−|Fi∩Gj||Fk∩Gj|)
 MathType@MTEF@5@5@+=feaafiart1ev1aaatCvAUfKttLearuWrP9MDH5MBPbIqV92AaeXatLxBI9gBaebbnrfifHhDYfgasaacH8akY=wiFfYdH8Gipec8Eeeu0xXdbba9frFj0=OqFfea0dXdd9vqai=hGuQ8kuc9pgc9s8qqaq=dirpe0xb9q8qiLsFr0=vr0=vr0dc8meaabaqaciaacaGaaeqabaqabeGadaaakeaafaqaaeWabaaabaWaaabuaeaadaaeqbqaamaaqafabaWaaabuaeaacqGG8baFcqWGgbGrdaWgaaWcbaGaemyAaKgabeaakiabgMIihlabdEeahnaaBaaaleaacqWGQbGAaeqaaOGaeiiFaWNaeiiFaWNaemOray0aaSbaaSqaaiabdMgaPbqabaGccqGHPiYXcqWGhbWrdaWgaaWcbaGaemiBaWgabeaakiabcYha8jabcYha8jabdAeagnaaBaaaleaacqWGRbWAaeqaaOGaeyykICSaem4raC0aaSbaaSqaaiabdYgaSbqabaGccqGG8baFcqGG8baFcqWGgbGrdaWgaaWcbaGaem4AaSgabeaakiabgMIihlabdEeahnaaBaaaleaacqWGQbGAaeqaaOGaeiiFaWhaleaacqWGSbaBcqGHGjsUcqWGQbGAaeqaniabggHiLdaaleaacqWGRbWAcqGHGjsUcqWGPbqAaeqaniabggHiLdaaleaacqWGQbGAaeqaniabggHiLdaaleaacqWGPbqAaeqaniabggHiLdGccqGH9aqpaeaadaaeqbqaamaaqafabaGaeiiFaWNaemOray0aaSbaaSqaaiabdMgaPbqabaGccqGHPiYXcqWGhbWrdaWgaaWcbaGaemOAaOgabeaakiabcYha8bWcbaGaemOAaOgabeqdcqGHris5aaWcbaGaemyAaKgabeqdcqGHris5aOWaaabuaeaacqGG8baFcqWGgbGrdaWgaaWcbaGaem4AaSgabeaakiabgMIihlabdEeahnaaBaaaleaacqWGQbGAaeqaaOGaeiiFaWhaleaacqWGRbWAcqGHGjsUcqWGPbqAaeqaniabggHiLdGcdaaeqbqaaiabcYha8jabdAeagnaaBaaaleaacqWGPbqAaeqaaOGaeyykICSaem4raC0aaSbaaSqaaiabdYgaSbqabaGccqGG8baFcqGG8baFcqWGgbGrdaWgaaWcbaGaem4AaSgabeaakiabgMIihlabdEeahnaaBaaaleaacqWGSbaBaeqaaOGaeiiFaWhaleaacqWGSbaBcqGHGjsUcqWGQbGAaeqaniabggHiLdGccqGH9aqpaeaadaaeqbqaamaaqafabaGaeiiFaWNaemOray0aaSbaaSqaaiabdMgaPbqabaGccqGHPiYXcqWGhbWrdaWgaaWcbaGaemOAaOgabeaakiabcYha8bWcbaGaemOAaOgabeqdcqGHris5aaWcbaGaemyAaKgabeqdcqGHris5aOWaaabuaeaacqGG8baFcqWGgbGrdaWgaaWcbaGaem4AaSgabeaakiabgMIihlabdEeahnaaBaaaleaacqWGQbGAaeqaaOGaeiiFaWhaleaacqWGRbWAcqGHGjsUcqWGPbqAaeqaniabggHiLdGcdaqadiqaamaaqafabaGaeiiFaWNaemOray0aaSbaaSqaaiabdMgaPbqabaGccqGHPiYXcqWGhbWrdaWgaaWcbaGaemiBaWgabeaakiabcYha8jabcYha8jabdAeagnaaBaaaleaacqWGRbWAaeqaaOGaeyykICSaem4raC0aaSbaaSqaaiabdYgaSbqabaGccqGG8baFaSqaaiabdYgaSbqab0GaeyyeIuoakiabgkHiTiabcYha8jabdAeagnaaBaaaleaacqWGPbqAaeqaaOGaeyykICSaem4raC0aaSbaaSqaaiabdQgaQbqabaGccqGG8baFcqGG8baFcqWGgbGrdaWgaaWcbaGaem4AaSgabeaakiabgMIihlabdEeahnaaBaaaleaacqWGQbGAaeqaaOGaeiiFaWhacaGLOaGaayzkaaaaaaaa@F676@

Inspired by the precomputing of sums used in shared(*v*, *v'*), (5), we calculate for each *i*, *k*, *k *≠ *i *the sum:

Si,k=∑l|Fi∩Gl||Fk∩Gl|     (11)
 MathType@MTEF@5@5@+=feaafiart1ev1aaatCvAUfKttLearuWrP9MDH5MBPbIqV92AaeXatLxBI9gBaebbnrfifHhDYfgasaacH8akY=wiFfYdH8Gipec8Eeeu0xXdbba9frFj0=OqFfea0dXdd9vqai=hGuQ8kuc9pgc9s8qqaq=dirpe0xb9q8qiLsFr0=vr0=vr0dc8meaabaqaciaacaGaaeqabaqabeGadaaakeaacqWGtbWudaWgaaWcbaGaemyAaKMaeiilaWIaem4AaSgabeaakiabg2da9maaqafabaGaeiiFaWNaemOray0aaSbaaSqaaiabdMgaPbqabaGccqGHPiYXcqWGhbWrdaWgaaWcbaGaemiBaWgabeaakiabcYha8bWcbaGaemiBaWgabeqdcqGHris5aOGaeiiFaWNaemOray0aaSbaaSqaaiabdUgaRbqabaGccqGHPiYXcqWGhbWrdaWgaaWcbaGaemiBaWgabeaakiabcYha8jaaxMaacaWLjaWaaeWaceaacqaIXaqmcqaIXaqmaiaawIcacaGLPaaaaaa@4ED6@

There are *O*(idv2
 MathType@MTEF@5@5@+=feaafiart1ev1aaatCvAUfKttLearuWrP9MDH5MBPbIqV92AaeXatLxBI9gBaebbnrfifHhDYfgasaacH8akY=wiFfYdH8Gipec8Eeeu0xXdbba9frFj0=OqFfea0dXdd9vqai=hGuQ8kuc9pgc9s8qqaq=dirpe0xb9q8qiLsFr0=vr0=vr0dc8meaabaqaciaacaGaaeqabaqabeGadaaakeaacqWGPbqAcqWGKbazdaqhaaWcbaGaemODayhabaGaeGOmaidaaaaa@31EC@) of these sums and each takes time *O*(*id*_*v'*_) to calculate, so the time complexity for calculating all sums is *O*(idv2idv′
 MathType@MTEF@5@5@+=feaafiart1ev1aaatCvAUfKttLearuWrP9MDH5MBPbIqV92AaeXatLxBI9gBaebbnrfifHhDYfgasaacH8akY=wiFfYdH8Gipec8Eeeu0xXdbba9frFj0=OqFfea0dXdd9vqai=hGuQ8kuc9pgc9s8qqaq=dirpe0xb9q8qiLsFr0=vr0=vr0dc8meaabaqaciaacaGaaeqabaqabeGadaaakeaacqWGPbqAcqWGKbazdaqhaaWcbaGaemODayhabaGaeGOmaidaaOGaemyAaKMaemizaq2aaSbaaSqaaiqbdAha2zaafaaabeaaaaa@364F@). In the case that *id*_*v' *_≤ *id*_*v*_, we can switch *v *and *v' *and thus get time usage *O*(*id*_*v*_*id*_*v' *_min{*id*_*v*_, *id*_*v'*_}). Assuming that the sums have been calculated, (9) can now be calculated in time *O*(*id*_*v*_*id*_*v'*_min{*id*_*v*_, *id*_*v'*_}) by the expression:

∑i∑j|Fi∩Gj|∑k≠i|Fk∩Gj|(Si,k−|Fi∩Gj||Fk∩Gj|)     (12)
 MathType@MTEF@5@5@+=feaafiart1ev1aaatCvAUfKttLearuWrP9MDH5MBPbIqV92AaeXatLxBI9gBaebbnrfifHhDYfgasaacH8akY=wiFfYdH8Gipec8Eeeu0xXdbba9frFj0=OqFfea0dXdd9vqai=hGuQ8kuc9pgc9s8qqaq=dirpe0xb9q8qiLsFr0=vr0=vr0dc8meaabaqaciaacaGaaeqabaqabeGadaaakeaadaaeqbqaamaaqafabaGaeiiFaWNaemOray0aaSbaaSqaaiabdMgaPbqabaGccqGHPiYXcqWGhbWrdaWgaaWcbaGaemOAaOgabeaakiabcYha8bWcbaGaemOAaOgabeqdcqGHris5aaWcbaGaemyAaKgabeqdcqGHris5aOWaaabuaeaacqGG8baFcqWGgbGrdaWgaaWcbaGaem4AaSgabeaakiabgMIihlabdEeahnaaBaaaleaacqWGQbGAaeqaaOGaeiiFaW3aaeWaceaacqWGtbWudaWgaaWcbaGaemyAaKMaeiilaWIaem4AaSgabeaakiabgkHiTiabcYha8jabdAeagnaaBaaaleaacqWGPbqAaeqaaOGaeyykICSaem4raC0aaSbaaSqaaiabdQgaQbqabaGccqGG8baFcqGG8baFcqWGgbGrdaWgaaWcbaGaem4AaSgabeaakiabgMIihlabdEeahnaaBaaaleaacqWGQbGAaeqaaOGaeiiFaWhacaGLOaGaayzkaaaaleaacqWGRbWAcqGHGjsUcqWGPbqAaeqaniabggHiLdGccaWLjaGaaCzcamaabmGabaGaeGymaeJaeGOmaidacaGLOaGaayzkaaaaaa@6E4A@

By substituting (9) with (12) in (10), we can calculate nonshared(*v*, *v'*, in time *O*(*id*_*v*_*id*_*v'*_min{*id*_*v*_*, id*_*v'*_}). Since calculation of the sums is independent of the calculation of nonshared(*v*, *v'*), these calculations can be done serially as shown in the algorithm below.

ALGORITHM – CALCULATING THE NUMBER OF NONSHARED BUTTERFLY QUARTETS BETWEEN *T *AND *T'*

**Requires: ***T*, *T' *two input trees with the same leaf set.

**Ensures: ***Res *= nonshared(*T*, *T'*)

*Res *← 0

**for ***v *internal node in *T ***do**

   **for ***v' *internal node in *T' ***do**

      Calculate sums S_*i*,*k*_

      *Res *← *Res *+ nonshared(*v*, *v'*)

   **end for**

end for

*Res *← Res4
 MathType@MTEF@5@5@+=feaafiart1ev1aaatCvAUfKttLearuWrP9MDH5MBPbIqV92AaeXatLxBI9gBaebbnrfifHhDYfgasaacH8akY=wiFfYdH8Gipec8Eeeu0xXdbba9frFj0=OqFfea0dXdd9vqai=hGuQ8kuc9pgc9s8qqaq=dirpe0xb9q8qiLsFr0=vr0=vr0dc8meaabaqaciaacaGaaeqabaqabeGadaaakeaadaWcaaqaaGqaciab=jfasjab=vgaLjab=nhaZbqaaiabisda0aaaaaa@31A0@

The time complexity of the algorithm is:

∑v∈T∑v′∈T′idvidv′min⁡{idv,idv′}≤min⁡{id,id′}∑v∈Tidv∑v′∈T′idv′=O(|V||V′|min⁡{id,id′})
 MathType@MTEF@5@5@+=feaafiart1ev1aaatCvAUfKttLearuWrP9MDH5MBPbIqV92AaeXatLxBI9gBaebbnrfifHhDYfgasaacH8akY=wiFfYdH8Gipec8Eeeu0xXdbba9frFj0=OqFfea0dXdd9vqai=hGuQ8kuc9pgc9s8qqaq=dirpe0xb9q8qiLsFr0=vr0=vr0dc8meaabaqaciaacaGaaeqabaqabeGadaaakeaadaaeqbqaamaaqafabaGaemyAaKMaemizaq2aaSbaaSqaaiabdAha2bqabaGccqWGPbqAcqWGKbazdaWgaaWcbaGafmODayNbauaaaeqaaOGagiyBa0MaeiyAaKMaeiOBa42aaiWabeaacqWGPbqAcqWGKbazdaWgaaWcbaGaemODayhabeaakiabcYcaSiabdMgaPjabdsgaKnaaBaaaleaacuWG2bGDgaqbaaqabaaakiaawUhacaGL9baacqGHKjYOcyGGTbqBcqGGPbqAcqGGUbGBdaGadeqaaiabdMgaPjabdsgaKjabcYcaSiabdMgaPjqbdsgaKzaafaaacaGL7bGaayzFaaWaaabuaeaacqWGPbqAcqWGKbazdaWgaaWcbaGaemODayhabeaakmaaqafabaGaemyAaKMaemizaq2aaSbaaSqaaiqbdAha2zaafaaabeaakiabg2da9iabd+eapnaabmGabaGaeiiFaWNaemOvayLaeiiFaWNaeiiFaWNafmOvayLbauaacqGG8baFcyGGTbqBcqGGPbqAcqGGUbGBdaGadeqaaiabdMgaPjabdsgaKjabcYcaSiabdMgaPjqbdsgaKzaafaaacaGL7bGaayzFaaaacaGLOaGaayzkaaaaleaacuWG2bGDgaqbaiabgIGiolqbdsfauzaafaaabeqdcqGHris5aaWcbaGaemODayNaeyicI4SaemivaqfabeqdcqGHris5aaWcbaGafmODayNbauaacqGHiiIZcuWGubavgaqbaaqab0GaeyyeIuoaaSqaaiabdAha2jabgIGiolabdsfaubqab0GaeyyeIuoaaaa@8E85@

### Counting butterfly quartets in a single tree

Reusing the idea of precomputing certain sums enables us to calculate the number of butterfly quartets in a single tree *T *in time *O*(|*V*|). Since the number of butterfly quartets in a single tree is the number of butterfly quartets shared between the tree and itself, we will use shared(*T*, *T*) to denote the number of butterfly quartet in *T*. This notation also emphasizes that computing the number is essentially a comparison of the tree against itself. Given a node *v *in *T *we can express the number of quartets it claims in the following way:

∑i(Fi2)((F¯i2)−∑j≠i(Fj2)),     (13)
 MathType@MTEF@5@5@+=feaafiart1ev1aaatCvAUfKttLearuWrP9MDH5MBPbIqV92AaeXatLxBI9gBaebbnrfifHhDYfgasaacH8akY=wiFfYdH8Gipec8Eeeu0xXdbba9frFj0=OqFfea0dXdd9vqai=hGuQ8kuc9pgc9s8qqaq=dirpe0xb9q8qiLsFr0=vr0=vr0dc8meaabaqaciaacaGaaeqabaqabeGadaaakeaadaaeqbqaamaabmGabaqbaeqabiqaaaqaaiabdAeagnaaBaaaleaacqWGPbqAaeqaaaGcbaGaeGOmaidaaaGaayjkaiaawMcaamaabmGabaWaaeWaceaafaqabeGabaaabaGafmOrayKbaebadaWgaaWcbaGaemyAaKgabeaaaOqaaiabikdaYaaaaiaawIcacaGLPaaacqGHsisldaaeqbqaamaabmGabaqbaeqabiqaaaqaaiabdAeagnaaBaaaleaacqWGQbGAaeqaaaGcbaGaeGOmaidaaaGaayjkaiaawMcaaaWcbaGaemOAaOMaeyiyIKRaemyAaKgabeqdcqGHris5aaGccaGLOaGaayzkaaaaleaacqWGPbqAaeqaniabggHiLdGccqGGSaalcaWLjaGaaCzcamaabmGabaGaeGymaeJaeG4mamdacaGLOaGaayzkaaaaaa@4E95@

where the *F*_*i*_'s are the subtrees of *v*. Now let

S=∑j(Fj2),
 MathType@MTEF@5@5@+=feaafiart1ev1aaatCvAUfKttLearuWrP9MDH5MBPbIqV92AaeXatLxBI9gBaebbnrfifHhDYfgasaacH8akY=wiFfYdH8Gipec8Eeeu0xXdbba9frFj0=OqFfea0dXdd9vqai=hGuQ8kuc9pgc9s8qqaq=dirpe0xb9q8qiLsFr0=vr0=vr0dc8meaabaqaciaacaGaaeqabaqabeGadaaakeaacqWGtbWucqGH9aqpdaaeqbqaamaabmGabaqbaeqabiqaaaqaaiabdAeagnaaBaaaleaacqWGQbGAaeqaaaGcbaGaeGOmaidaaaGaayjkaiaawMcaaaWcbaGaemOAaOgabeqdcqGHris5aOGaeiilaWcaaa@387D@

we can now express (13) as

∑i(Fi2)((F¯i2)−S+(Fi2))⋅     (14)
 MathType@MTEF@5@5@+=feaafiart1ev1aaatCvAUfKttLearuWrP9MDH5MBPbIqV92AaeXatLxBI9gBaebbnrfifHhDYfgasaacH8akY=wiFfYdH8Gipec8Eeeu0xXdbba9frFj0=OqFfea0dXdd9vqai=hGuQ8kuc9pgc9s8qqaq=dirpe0xb9q8qiLsFr0=vr0=vr0dc8meaabaqaciaacaGaaeqabaqabeGadaaakeaadaaeqbqaamaabmGabaqbaeqabiqaaaqaaiabdAeagnaaBaaaleaacqWGPbqAaeqaaaGcbaGaeGOmaidaaaGaayjkaiaawMcaamaabmGabaWaaeWaceaafaqabeGabaaabaGafmOrayKbaebadaWgaaWcbaGaemyAaKgabeaaaOqaaiabikdaYaaaaiaawIcacaGLPaaacqGHsislcqWGtbWucqGHRaWkdaqadiqaauaabeqaceaaaeaacqWGgbGrdaWgaaWcbaGaemyAaKgabeaaaOqaaiabikdaYaaaaiaawIcacaGLPaaaaiaawIcacaGLPaaaaSqaaiabdMgaPbqab0GaeyyeIuoakiabgwSixlaaxMaacaWLjaWaaeWaceaacqaIXaqmcqaI0aanaiaawIcacaGLPaaaaaa@4B64@

*S *can be calculated in time *O*(*id*_*v*_)and using the precomputed *S*, (14) can be also calculated in time *O*(*id*_*v*_). Summing the results of (14) for all nodes in *T *gives the number of quartets in the tree, shared(*T*, *T*). The total time usage is

∑v∈Tidv=O(|V|).
 MathType@MTEF@5@5@+=feaafiart1ev1aaatCvAUfKttLearuWrP9MDH5MBPbIqV92AaeXatLxBI9gBaebbnrfifHhDYfgasaacH8akY=wiFfYdH8Gipec8Eeeu0xXdbba9frFj0=OqFfea0dXdd9vqai=hGuQ8kuc9pgc9s8qqaq=dirpe0xb9q8qiLsFr0=vr0=vr0dc8meaabaqaciaacaGaaeqabaqabeGadaaakeaadaaeqbqaaiabdMgaPjabdsgaKnaaBaaaleaacqWG2bGDaeqaaaqaaiabdAha2jabgIGiolabdsfaubqab0GaeyyeIuoakiabg2da9iabd+eapnaabmGabaGaeiiFaWNaemOvayLaeiiFaWhacaGLOaGaayzkaaGaeiOla4caaa@4016@

### Calculating the shared leaf set sizes

The algorithms presented above all rely on *O*(1) time access to the size of the shared leaf set |*F *∩ *G*| for any pair of subtrees *F *of *T *and *G *of *T'*, where *F *and *G *each has size at least two, i.e. contains more than a single leaf. We will refer to |*F *∩ *G*| as the intersection size of subtree *F *and *G*. In [9] and *O*(*n*^2^) time and space algorithm is presented for computing the intersection sizes of all pairs of subtrees *F *and *G *of two binary input trees. A straightforward generalization of this algorithm to two input trees *T *and *T' *of arbitrary degrees results in an *O*(*n*^2^*dd'*) time and *O*(*n*^2^) space algorithm, which gives a worst case running time of *O*(*n*^4^).

**Figure 10:**
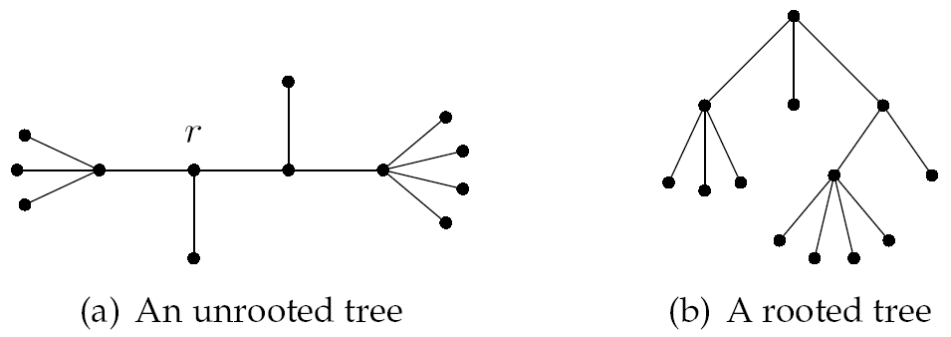
An arbitrary node, *r*, is chosen as the root in the tree, leading to a rooted tree.

In this section we will present an improved algorithm for computing the intersection sizes of all pairs of subtrees of *T *and *T' *which runs in time *O*(*n *+ |*V*||*V'*|) and space *O*(|*V*||*V'*|). We will assume that the size of each subtree *F *of *T *and *G *of *T'*, i.e. |*F*| and |*G*|, is available in time *O*(1). This can be achieved as presented in the next section. Our algorithm for computing all intersection sizes is as follows. Choose an arbitrary node *r *in *T *and an arbitrary node *r' *in *T'*. Rooting the trees in *r *and *r' *respectively gives rise to two rooted trees *T*_*r *_and T′r
 MathType@MTEF@5@5@+=feaafiart1ev1aaatCvAUfKttLearuWrP9MDH5MBPbIqV92AaeXatLxBI9gBaebbnrfifHhDYfgasaacH8akY=wiFfYdH8Gipec8Eeeu0xXdbba9frFj0=OqFfea0dXdd9vqai=hGuQ8kuc9pgc9s8qqaq=dirpe0xb9q8qiLsFr0=vr0=vr0dc8meaabaqaciaacaGaaeqabaqabeGadaaakeaacuWGubavgaqbamaaBaaaleaacqWGYbGCaeqaaaaa@2F82@, Fig. 10 shows an example of rooting a tree. Calculating the shared leaf set sizes of *T*_*r *_and T′r
 MathType@MTEF@5@5@+=feaafiart1ev1aaatCvAUfKttLearuWrP9MDH5MBPbIqV92AaeXatLxBI9gBaebbnrfifHhDYfgasaacH8akY=wiFfYdH8Gipec8Eeeu0xXdbba9frFj0=OqFfea0dXdd9vqai=hGuQ8kuc9pgc9s8qqaq=dirpe0xb9q8qiLsFr0=vr0=vr0dc8meaabaqaciaacaGaaeqabaqabeGadaaakeaacuWGubavgaqbamaaBaaaleaacqWGYbGCaeqaaaaa@2F82@ and all subtrees in both trees can be done using:

|Tr∩T′r|=∑i∑j|Fi∩Gj|=∑i|Fi∩G|=∑j|F∩Gj|,     (15)
 MathType@MTEF@5@5@+=feaafiart1ev1aaatCvAUfKttLearuWrP9MDH5MBPbIqV92AaeXatLxBI9gBaebbnrfifHhDYfgasaacH8akY=wiFfYdH8Gipec8Eeeu0xXdbba9frFj0=OqFfea0dXdd9vqai=hGuQ8kuc9pgc9s8qqaq=dirpe0xb9q8qiLsFr0=vr0=vr0dc8meaabaqaciaacaGaaeqabaqabeGadaaakeaacqGG8baFcqWGubavdaWgaaWcbaGaemOCaihabeaakiabgMIihlqbdsfauzaafaWaaSbaaSqaaiabdkhaYbqabaGccqGG8baFcqGH9aqpdaaeqbqaamaaqafabaGaeiiFaWNaemOray0aaSbaaSqaaiabdMgaPbqabaGccqGHPiYXcqWGhbWrdaWgaaWcbaGaemOAaOgabeaakiabcYha8jabg2da9maaqafabaGaeiiFaWNaemOray0aaSbaaSqaaiabdMgaPbqabaGccqGHPiYXcqWGhbWrcqGG8baFcqGH9aqpdaaeqbqaaiabcYha8jabdAeagjabgMIihlabdEeahnaaBaaaleaacqWGQbGAaeqaaOGaeiiFaWNaeiilaWcaleaacqWGQbGAaeqaniabggHiLdaaleaacqWGPbqAaeqaniabggHiLdaaleaacqWGQbGAaeqaniabggHiLdaaleaacqWGPbqAaeqaniabggHiLdGccaWLjaGaaCzcamaabmGabaGaeGymaeJaeGynaudacaGLOaGaayzkaaaaaa@6853@

where *F*_*i *_are all subtrees of *T*_*r *_and *G*_*j *_are all subtrees of T′r
 MathType@MTEF@5@5@+=feaafiart1ev1aaatCvAUfKttLearuWrP9MDH5MBPbIqV92AaeXatLxBI9gBaebbnrfifHhDYfgasaacH8akY=wiFfYdH8Gipec8Eeeu0xXdbba9frFj0=OqFfea0dXdd9vqai=hGuQ8kuc9pgc9s8qqaq=dirpe0xb9q8qiLsFr0=vr0=vr0dc8meaabaqaciaacaGaaeqabaqabeGadaaakeaacuWGubavgaqbamaaBaaaleaacqWGYbGCaeqaaaaa@2F82@. This can be calculated using dynamic programming in time *O*(*n*^2^):

∑v∈V∑v′∈V′dvdv′+∑v∈V∑l′∈L′dv+∑l∈L∑v′∈V′dv′+∑l∈L∑l′∈L′1=O(n2)
 MathType@MTEF@5@5@+=feaafiart1ev1aaatCvAUfKttLearuWrP9MDH5MBPbIqV92AaeXatLxBI9gBaebbnrfifHhDYfgasaacH8akY=wiFfYdH8Gipec8Eeeu0xXdbba9frFj0=OqFfea0dXdd9vqai=hGuQ8kuc9pgc9s8qqaq=dirpe0xb9q8qiLsFr0=vr0=vr0dc8meaabaqaciaacaGaaeqabaqabeGadaaakeaadaaeqbqaamaaqafabaGaemizaq2aaSbaaSqaaiabdAha2bqabaGccqWGKbazdaWgaaWcbaGafmODayNbauaaaeqaaOGaey4kaSYaaabuaeaadaaeqbqaaiabdsgaKnaaBaaaleaacqWG2bGDaeqaaOGaey4kaSYaaabuaeaadaaeqbqaaiabdsgaKnaaBaaaleaacuWG2bGDgaqbaaqabaaabaGafmODayNbauaacqGHiiIZcuWGwbGvgaqbaaqab0GaeyyeIuoaaSqaaiabdYgaSjabgIGiolabdYeambqab0GaeyyeIuoakiabgUcaRmaaqafabaWaaabuaeaacqaIXaqmcqGH9aqpcqWGpbWtdaqadiqaaiabd6gaUnaaCaaaleqabaGaeGOmaidaaaGccaGLOaGaayzkaaaaleaacuWGSbaBgaqbaiabgIGiolqbdYeamzaafaaabeqdcqGHris5aaWcbaGaemiBaWMaeyicI4SaemitaWeabeqdcqGHris5aaWcbaGafmiBaWMbauaacqGHiiIZcuWGmbatgaqbaaqab0GaeyyeIuoaaSqaaiabdAha2jabgIGiolabdAfawbqab0GaeyyeIuoaaSqaaiqbdAha2zaafaGaeyicI4SafmOvayLbauaaaeqaniabggHiLdaaleaacqWG2bGDcqGHiiIZcqWGwbGvaeqaniabggHiLdaaaa@74CD@

Except |*T*_*r *_∩ T′r
 MathType@MTEF@5@5@+=feaafiart1ev1aaatCvAUfKttLearuWrP9MDH5MBPbIqV92AaeXatLxBI9gBaebbnrfifHhDYfgasaacH8akY=wiFfYdH8Gipec8Eeeu0xXdbba9frFj0=OqFfea0dXdd9vqai=hGuQ8kuc9pgc9s8qqaq=dirpe0xb9q8qiLsFr0=vr0=vr0dc8meaabaqaciaacaGaaeqabaqabeGadaaakeaacuWGubavgaqbamaaBaaaleaacqWGYbGCaeqaaaaa@2F82@|, the shared leafset sizes calculated by (15) are the shared leaf set sizes of all rooted subtrees of *T *and *T' *that do not contain the nodes *r *and *r'*. Assuming that the subtree *F *of *T *does *not *contain *r*, then the subtree F¯
 MathType@MTEF@5@5@+=feaafiart1ev1aaatCvAUfKttLearuWrP9MDH5MBPbIqV92AaeXatLxBI9gBaebbnrfifHhDYfgasaacH8akY=wiFfYdH8Gipec8Eeeu0xXdbba9frFj0=OqFfea0dXdd9vqai=hGuQ8kuc9pgc9s8qqaq=dirpe0xb9q8qiLsFr0=vr0=vr0dc8meaabaqaciaacaGaaeqabaqabeGadaaakeaacuWGgbGrgaqeaaaa@2DD9@*does *contain *r *and similarly for *r' *and subtrees *G *and G¯
 MathType@MTEF@5@5@+=feaafiart1ev1aaatCvAUfKttLearuWrP9MDH5MBPbIqV92AaeXatLxBI9gBaebbnrfifHhDYfgasaacH8akY=wiFfYdH8Gipec8Eeeu0xXdbba9frFj0=OqFfea0dXdd9vqai=hGuQ8kuc9pgc9s8qqaq=dirpe0xb9q8qiLsFr0=vr0=vr0dc8meaabaqaciaacaGaaeqabaqabeGadaaakeaacuWGhbWrgaqeaaaa@2DDB@ of *T*'. The shared leaf set sizes of these trees that *do *contain *r *and *r' *can be calculated from the intersection sizes that we have available using (16):

|F¯∩G|=|G|−|F∩G||F∩G¯|=|F|−|F∩G||F¯∩G¯|=n−(|G|+|F|−|F∩G|)     (16)
 MathType@MTEF@5@5@+=feaafiart1ev1aaatCvAUfKttLearuWrP9MDH5MBPbIqV92AaeXatLxBI9gBaebbnrfifHhDYfgasaacH8akY=wiFfYdH8Gipec8Eeeu0xXdbba9frFj0=OqFfea0dXdd9vqai=hGuQ8kuc9pgc9s8qqaq=dirpe0xb9q8qiLsFr0=vr0=vr0dc8meaabaqaciaacaGaaeqabaqabeGadaaakeaafaqaaeWadaaabaGaeiiFaWNafmOrayKbaebacqGHPiYXcqWGhbWrcqGG8baFaeaacqGH9aqpaeaacqGG8baFcqWGhbWrcqGG8baFcqGHsislcqGG8baFcqWGgbGrcqGHPiYXcqWGhbWrcqGG8baFaeaacqGG8baFcqWGgbGrcqGHPiYXcuWGhbWrgaqeaiabcYha8bqaaiabg2da9aqaaiabcYha8jabdAeagjabcYha8jabgkHiTiabcYha8jabdAeagjabgMIihlabdEeahjabcYha8bqaaiabcYha8jqbdAeagzaaraGaeyykICSafm4raCKbaebacqGG8baFaeaacqGH9aqpaeaacqWGUbGBcqGHsisldaqadiqaaiabcYha8jabdEeahjabcYha8jabgUcaRiabcYha8jabdAeagjabcYha8jabgkHiTiabcYha8jabdAeagjabgMIihlabdEeahjabcYha8bGaayjkaiaawMcaaaaacaWLjaGaaCzcamaabmGabaGaeGymaeJaeGOnaydacaGLOaGaayzkaaaaaa@7587@

In other words all shared leaf set sizes can be calculated in time *O*(*n*^2^). First the shared leaf set sizes for subtrees that to not contain an arbitrary node *r *and *r' *from each tree are calculated in time *O*(*n*^2^). Then the shared leaf set sizes of subtrees that do contain *r *or *r' *(or both) are calculated constant time for each shared leaf set. Since there are *O*(*n*^2^) shared leaf sets the total time usage is *O*(*n*^2^).

The reduction to time *O*(*n *+ |*V*||*V'*|) and space *O*(|*V*||*V'*|) is done by handling the cases where *F*, *F*_*i*_, *G *or *G*_*j *_is a leaf in a special way. For each pair of nodes *v*, *v' *we let *Leaf *[*v*, *v'*] be the number of leaves directly connected to *v *that have the same label as a leaf directly connected to *v'. Leaf *[*v*, *v'*] is constructable in time *O*(*n *+ |*V||*|*V*'|) in the following way: First, set *Leaf *[*v*, *v'*] = 0 for all pairs of nodes *v*, *v'*. Given a leaf number, *x*, there is a unique node, *node*(*x*), in *T *and a unique node, *node'*(*x*), in *T'*. For each leaf number, *x*, we increment *Leaf *[*node*(*x*)*, node'*(*x*)]. There are *n *such numbers, and by assumption, the leaves can be found in constant time given a number. Thus *Leaf *[*v*, *v'*] is constructable in time *O*(*n *+ |*V*||*V'*|). We choose *r *and *r' *in *T *and *T' *and create two rooted trees *T*_*r *_and T′r
 MathType@MTEF@5@5@+=feaafiart1ev1aaatCvAUfKttLearuWrP9MDH5MBPbIqV92AaeXatLxBI9gBaebbnrfifHhDYfgasaacH8akY=wiFfYdH8Gipec8Eeeu0xXdbba9frFj0=OqFfea0dXdd9vqai=hGuQ8kuc9pgc9s8qqaq=dirpe0xb9q8qiLsFr0=vr0=vr0dc8meaabaqaciaacaGaaeqabaqabeGadaaakeaacuWGubavgaqbamaaBaaaleaacqWGYbGCaeqaaaaa@2F82@. The *non-leaf *children of *r *in *T*_*r *_are *F*_1_,..., *F*_*x *_and the *non-leaf *children of *r' *in T′r
 MathType@MTEF@5@5@+=feaafiart1ev1aaatCvAUfKttLearuWrP9MDH5MBPbIqV92AaeXatLxBI9gBaebbnrfifHhDYfgasaacH8akY=wiFfYdH8Gipec8Eeeu0xXdbba9frFj0=OqFfea0dXdd9vqai=hGuQ8kuc9pgc9s8qqaq=dirpe0xb9q8qiLsFr0=vr0=vr0dc8meaabaqaciaacaGaaeqabaqabeGadaaakeaacuWGubavgaqbamaaBaaaleaacqWGYbGCaeqaaaaa@2F82@ are *G*_1_,..., *G*_*y*_. The intersection size of the two trees can be defined recursively as:

|Tr∩T′r|=Leaf[r,r′]+∑i=1x|Fi∩T′r|+∑j=1y|Tr∩Gj|−∑i=1x∑j=1y|Fi∩Gj|     (17)
 MathType@MTEF@5@5@+=feaafiart1ev1aaatCvAUfKttLearuWrP9MDH5MBPbIqV92AaeXatLxBI9gBaebbnrfifHhDYfgasaacH8akY=wiFfYdH8Gipec8Eeeu0xXdbba9frFj0=OqFfea0dXdd9vqai=hGuQ8kuc9pgc9s8qqaq=dirpe0xb9q8qiLsFr0=vr0=vr0dc8meaabaqaciaacaGaaeqabaqabeGadaaakeaacqGG8baFcqWGubavdaWgaaWcbaGaemOCaihabeaakiabgMIihlqbdsfauzaafaWaaSbaaSqaaiabdkhaYbqabaGccqGG8baFcqGH9aqpcqWGmbatcqWGLbqzcqWGHbqycqWGMbGzdaWadiqaaiabdkhaYjabcYcaSiqbdkhaYzaafaaacaGLBbGaayzxaaGaey4kaSYaaabCaeaacqGG8baFcqWGgbGrdaWgaaWcbaGaemyAaKgabeaakiabgMIihlqbdsfauzaafaWaaSbaaSqaaiabdkhaYbqabaGccqGG8baFaSqaaiabdMgaPjabg2da9iabigdaXaqaaiabdIha4bqdcqGHris5aOGaey4kaSYaaabCaeaacqGG8baFcqWGubavdaWgaaWcbaGaemOCaihabeaakiabgMIihlabdEeahnaaBaaaleaacqWGQbGAaeqaaOGaeiiFaWNaeyOeI0YaaabCaeaadaaeWbqaaiabcYha8jabdAeagnaaBaaaleaacqWGPbqAaeqaaOGaeyykICSaem4raC0aaSbaaSqaaiabdQgaQbqabaGccqGG8baFaSqaaiabdQgaQjabg2da9iabigdaXaqaaiabdMha5bqdcqGHris5aaWcbaGaemyAaKMaeyypa0JaeGymaedabaGaemiEaGhaniabggHiLdaaleaacqWGQbGAcqGH9aqpcqaIXaqmaeaacqWG5bqEa0GaeyyeIuoakiaaxMaacaWLjaWaaeWaceaacqaIXaqmcqaI3aWnaiaawIcacaGLPaaaaaa@84B8@

The first term counts all leaves directly connected to both *r *and *r'*. The second term counts all leaves connected directly to *r'*, that are also in *T*_*r*_, but not directly connected to *r*. The third term counts all leaves connected directly to *r*, that are also in T′r
 MathType@MTEF@5@5@+=feaafiart1ev1aaatCvAUfKttLearuWrP9MDH5MBPbIqV92AaeXatLxBI9gBaebbnrfifHhDYfgasaacH8akY=wiFfYdH8Gipec8Eeeu0xXdbba9frFj0=OqFfea0dXdd9vqai=hGuQ8kuc9pgc9s8qqaq=dirpe0xb9q8qiLsFr0=vr0=vr0dc8meaabaqaciaacaGaaeqabaqabeGadaaakeaacuWGubavgaqbamaaBaaaleaacqWGYbGCaeqaaaaa@2F82@, but not directly connected to *r'*. Summing these three terms counts all leaves present in both subtrees, but leaves not connected directly to the roots are counted twice, and are subtracted by the last term.

Since (17) is only summing over the *non-leaf *children of a given internal node, calculating the shared leaf set sizes of all these pairs of subtrees can be done using dynamic programming in time:

(n+|V||V′|)+∑v∈Vidv+∑v′∈V′idv′+∑v∈V∑v′∈V′idvidv′=O(n+|V||V′|)
 MathType@MTEF@5@5@+=feaafiart1ev1aaatCvAUfKttLearuWrP9MDH5MBPbIqV92AaeXatLxBI9gBaebbnrfifHhDYfgasaacH8akY=wiFfYdH8Gipec8Eeeu0xXdbba9frFj0=OqFfea0dXdd9vqai=hGuQ8kuc9pgc9s8qqaq=dirpe0xb9q8qiLsFr0=vr0=vr0dc8meaabaqaciaacaGaaeqabaqabeGadaaakeaadaqadiqaaiabd6gaUjabgUcaRiabcYha8jabdAfawjabcYha8jabcYha8jqbdAfawzaafaGaeiiFaWhacaGLOaGaayzkaaGaey4kaSYaaabuaeaacqWGPbqAcqWGKbazdaWgaaWcbaGaemODayhabeaakiabgUcaRmaaqafabaGaemyAaKMaemizaq2aaSbaaSqaaiqbdAha2zaafaaabeaaaeaacuWG2bGDgaqbaiabgIGiolqbdAfawzaafaaabeqdcqGHris5aOGaey4kaSYaaabuaeaadaaeqbqaaiabdMgaPjabdsgaKnaaBaaaleaacqWG2bGDaeqaaOGaemyAaKMaemizaq2aaSbaaSqaaiqbdAha2zaafaaabeaakiabg2da9iabd+eapnaabmGabaGaemOBa4Maey4kaSIaeiiFaWNaemOvayLaeiiFaWNaeiiFaWNafmOvayLbauaacqGG8baFaiaawIcacaGLPaaaaSqaaiqbdAha2zaafaGaeyicI4SafmOvayLbauaaaeqaniabggHiLdaaleaacqWG2bGDcqGHiiIZcqWGwbGvaeqaniabggHiLdaaleaacqWG2bGDcqGHiiIZcqWGwbGvaeqaniabggHiLdaaaa@74EC@

By the same arguments as above, the rest of the shared leaf set sizes can be computed in time *O*(|*V*||*V'*|) and space *O*(|*V*||*V'*|). Therefore the total running time of the algorithm is *O*(*n *+ |*V*||*V'*|) and space usage is *O*(|*V*||*V'*|).

### Calculating the sizes of all subtree leaf sets

All of the above algorithms make use of the sizes of the leaf sets of the rooted subtrees of the input trees, either directly or indirectly. Rooting *T *in an arbitrary node *r *gives rise to the rooted tree *T*_*r*_. Every subtree *F*_*x *_of *T*_*r *_is a rooted subtree of *T*, and F¯
 MathType@MTEF@5@5@+=feaafiart1ev1aaatCvAUfKttLearuWrP9MDH5MBPbIqV92AaeXatLxBI9gBaebbnrfifHhDYfgasaacH8akY=wiFfYdH8Gipec8Eeeu0xXdbba9frFj0=OqFfea0dXdd9vqai=hGuQ8kuc9pgc9s8qqaq=dirpe0xb9q8qiLsFr0=vr0=vr0dc8meaabaqaciaacaGaaeqabaqabeGadaaakeaacuWGgbGrgaqeaaaa@2DD9@_*x *_is also a rooted subtree of *T*. Note that the set of subtrees *F*_*x *_∪ F¯
 MathType@MTEF@5@5@+=feaafiart1ev1aaatCvAUfKttLearuWrP9MDH5MBPbIqV92AaeXatLxBI9gBaebbnrfifHhDYfgasaacH8akY=wiFfYdH8Gipec8Eeeu0xXdbba9frFj0=OqFfea0dXdd9vqai=hGuQ8kuc9pgc9s8qqaq=dirpe0xb9q8qiLsFr0=vr0=vr0dc8meaabaqaciaacaGaaeqabaqabeGadaaakeaacuWGgbGrgaqeaaaa@2DD9@_*x*_, since one tree is the complement of the other, contains all subtrees of *T *and that |F¯
 MathType@MTEF@5@5@+=feaafiart1ev1aaatCvAUfKttLearuWrP9MDH5MBPbIqV92AaeXatLxBI9gBaebbnrfifHhDYfgasaacH8akY=wiFfYdH8Gipec8Eeeu0xXdbba9frFj0=OqFfea0dXdd9vqai=hGuQ8kuc9pgc9s8qqaq=dirpe0xb9q8qiLsFr0=vr0=vr0dc8meaabaqaciaacaGaaeqabaqabeGadaaakeaacuWGgbGrgaqeaaaa@2DD9@_*x*_| = *n *- |*F*_*x*_|. By using dynamic programming the sizes of all subtrees, *F*_*x*_, can be computed by a single traversal of *T*_*r*_. For each *F*_*x *_the size of F¯
 MathType@MTEF@5@5@+=feaafiart1ev1aaatCvAUfKttLearuWrP9MDH5MBPbIqV92AaeXatLxBI9gBaebbnrfifHhDYfgasaacH8akY=wiFfYdH8Gipec8Eeeu0xXdbba9frFj0=OqFfea0dXdd9vqai=hGuQ8kuc9pgc9s8qqaq=dirpe0xb9q8qiLsFr0=vr0=vr0dc8meaabaqaciaacaGaaeqabaqabeGadaaakeaacuWGgbGrgaqeaaaa@2DD9@_*x *_can be computed in constant time, since *n *is known. This means that all leaf set sizes of a tree of arbitrary degree can be calculated in time *O*(*n*).

## Authors' contributions

All authors participated in developing the algorithm. CC and MR implemented the algorithm and conducted the experiments. All authors participated in drafting of the manuscript.

## References

[B1] Felsenstein J (2004). Inferring Phylogenies.

[B2] Robinson DP, Foulds LR (1979). Comparison of weighted labelled trees. Combinatorial mathematics, VI (Proc 6th Austral Conf).

[B3] Waterman MS, Smith TF (1978). On the similarity of dendrograms. Journal of Theoretical Biology.

[B4] Allen BL, Steel M (2001). Subtree transfer operations and their induced metrics on evolutionary trees. Annals of Combinatorics.

[B5] Robinson DP, Foulds LR (1981). Comparison of phylogenetic trees. Mathematical Biosciences.

[B6] Estabrook G, McMorris F, Meacham C (1985). Comparison of undirected phylogenetic trees based on subtrees of four evolutionary units. Syst Zool.

[B7] Steel M, Penny D (1993). Distribution of tree comparison metrics–some new results. Syst Biol.

[B8] Doucette CR (1985). An Efficient Algorithm to Compute Quartet Dissimilarity Measures.

[B9] Bryant D, Tsang J, Kearney PE, Li M (2000). Computing the quartet distance between evolutionary trees. Proceedings of the 11th Annual Symposium on Discrete Algorithms (SODA).

[B10] Brodal GS, Fagerberg R, Pedersen CNS (2003). Computing the Quartet Distance Between Evolutionary Trees in Time *O*(*n *log *n*). Algorithmica.

[B11] Christiansen C, Mailund T, Pedersen CNS, Randers M (2005). Computing the Quartet Distance Between Trees of Arbitrary Degree. Proceedings of Workshop on Algorithms in Bioinformatics (WABI).

[B12] r8s. http://ginger.ucdavis.edu/r8s/.

[B13] Pfam. http://www.sanger.ac.uk/Software/Pfam/.

[B14] Besenbacher S, Mailund T, Westh-Nielsen L, Pedersen CNS (2005). RBT – A tool for building refined Buneman trees. Bioinformatics.

[B15] QuartetDist. http://www.daimi.au.dk/~chrisc/qdist/.

